# ctDNA dynamics: a novel indicator to track resistance in metastatic breast cancer treated with anti-HER2 therapy

**DOI:** 10.18632/oncotarget.11791

**Published:** 2016-09-01

**Authors:** Fei Ma, Wenjie Zhu, Yanfang Guan, Ling Yang, Xuefeng Xia, Shanshan Chen, Qiao Li, Xiuwen Guan, Zongbi Yi, Haili Qian, Xin Yi, Binghe Xu

**Affiliations:** ^1^ Department of Medical Oncology, National Cancer Center/Cancer Hospital, Chinese Academy of Medical Sciences and Peking Union Medical College, Beijing, China; ^2^ Geneplus-Beijing, Beijing, China; ^3^ Houston Methodist Research Institute, Weill Cornell School of Medicine, Houston, TX, USA; ^4^ State Key Laboratory of Molecular Oncology, Cancer Institute/Hospital, Chinese Academy of Medical Sciences and Peking Union Medical College, Beijing, China

**Keywords:** circulating tumor DNA, dynamics, progression, metastatic breast cancer, anti-HER2 therapy

## Abstract

**Background:**

Most studies utilizing circulating tumor DNA (ctDNA) to monitor disease interrogated only one or a few genes and failed to develop workable criteria to inform clinical practice. We evaluated the feasibility of detecting resistance to anti-HER2 therapy by serial gene-panel ctDNA sequencing.

**Results:**

Primary therapeutic resistance was identified in 6 out of 14 patients with events of progressive disease. For this subset comparison of pre- and post-treatment ctDNA assay results revealed that *HER2* amplification concurred with disease progression (4/6, 66.7%). Mutations in *TP53* (3/6, 50.0%) and genes implicated in the PI3K/mTOR pathway (3/6, 50.0%) were also dominant markers of resistance. Together, resistance to HER2 blockade should be indicated during treatment if any of the following situations applies: 1) recurrence or persistence of *HER2* amplification in the blood; 2) emergence or ≥20% increase in the fraction of mutations in any of these resistance-related genes including *TP53/PIK3CA/MTOR/PTEN*. Compared with CT scans, dynamic ctDNA profiling utilizing pre-defined criteria was sensitive in identifying drug resistance (sensitivity 85.7%, specificity 55.0%), with a concordance rate up to 82.1%. Besides, the ctDNA criteria had a discriminating role in the prognosis of HER2-positive metastatic breast cancer.

**Methods:**

52 plasma samples were prospectively collected from 18 patients with HER2-positive metastatic breast cancer who were treated with an oral anti-HER1/HER2 tyrosine kinase inhibitor (ClinicalTrials.gov NCT01937689). ctDNA was assayed by gene-panel target-capture next-generation sequencing.

**Conclusions:**

Longitudinal gene-panel ctDNA sequencing could be exploited to determine resistance and guide the precise administration of anti-HER2 targeted therapy in the metastatic setting.

## INTRODUCTION

Metastatic breast cancer (MBC), which is largely incurable, poses a major challenge to the management of breast cancer. Human epidermal growth factor receptor 2 (HER2)-positive breast cancer accounts for approximately 20-30% of total breast cancer cases and is associated with inferior prognosis compared to HER2-negative subtypes [1–5]. Despite the established efficacy of standard anti-HER2 therapy and emerging therapeutic options in the breast medical oncology armamentarium, precise evaluation of response to treatment in the metastatic setting remains problematic in clinical practice. Serial assessment by radiological imaging may be inconclusive and fail to rapidly detect drug resistance. In addition, it is clinically crucial to identify resistance-conferring genomic events, particularly in heavily treated cases, so as to disclose actionable targets for subsequent therapy. Tumor genotyping by repeated tissue biopsies is subjected to spatial selection bias [6, 7] and precluded by complications associated with this procedure [8]. Hence, non-invasive biomarkers that can be utilized to monitor the disease in real-time and molecularly characterize drug resistance are urgently needed.

The clinical application of circulating tumor DNA (ctDNA) as a “liquid biopsy” has been investigated in recent years [9]. ctDNA, which carries tumor-specific genetic alterations, is shed by tumor cells into the bloodstream and represents only a small fraction of cell-free DNA (cfDNA) [10, 11]. ctDNA can be employed to monitor tumor dynamics in multiple malignancies including breast cancer [12, 13]. In a proof-of-concept study, Dawson et al evaluated ctDNA in serially collected blood samples from patients with MBC and determined that ctDNA exhibited greater correlation with changes in tumor burden than circulating tumor cells (CTCs) and CA15-3 [14]. Moreover, it is feasible to capture resistance to targeted therapy, either intrinsic or acquired, and determine the molecular basis by profiling ctDNA in cancer [15–18].

However, most studies incorporating ctDNA assay into disease monitoring interrogated only one or a few genes and failed to develop workable criteria to inform clinical practice. Multigene mechanisms have been implicated in resistance to HER2 blockade agents [19–22]. Hence we hypothesized that serial gene-panel sequencing of ctDNA would be sensitive and accurate in identifying therapeutic resistance. The current study was conducted in the setting of a prospective clinical trial which evaluated the safety and efficacy of pyrotinib in HER2-positive MBC [23]. Pyrotinib is a novel small molecule tyrosine kinase inhibitor (TKI) which has irreversible inhibitory capacity towards HER1 and HER2. Patients who had been previously exposed to anti-HER2 TKIs were excluded from the trial. Preliminary data showed its manageable toxicity and promising anti-tumor activity, with an overall objective response rate (ORR) of 52.8% and median progression-free survival (PFS) of 35.3 weeks [23]. We exploited ctDNA from serially collected samples to identify resistance-related tumor genetic alterations and made an initial attempt to establish ctDNA-based criteria to detect resistance to anti-HER2 therapy.

## RESULTS

### Patients and samples

In total, 18 patients (Supplementary Table S1) whose diseases were histologically confirmed as HER2 positive before enrollment were evaluated in the present study. The other participants of the clinical trial who did not give consent to sample collection were excluded from the current analysis. 52 prospectively collected plasma samples and temporally matched peripheral blood cells were assayed for somatic genomic alterations by target-capture next-generation sequencing (NGS). A panel of 368 genes was interrogated in the present study (Supplementary Table S2). The average sequencing coverage depth of 52 plasma samples was 704-fold, and the coverage rate of the target region was >99% (Supplementary Table S3).

### Identification of somatic genome alterations

We identified copy number variants (CNVs) in 17 out of 18 (94.4%) patients and in 33 of 52 (63.5%) plasma samples by analyzing the sequencing data for plasma matched with blood cells from the same patient (Supplementary Table S4). Amplification of the *ERBB2* gene, which encodes the HER2 protein, was predominant and identified in 13 of 18 (72.2%) patients and 20 of 52 (38.5%) plasma samples. In addition, ctDNA sequencing identified other less common CNVs in the study population. Elevated levels of *CDK12* were present in 6 of 52 plasma samples (11.5%), all of which were characterized by *ERBB2* and *CDK12* co-amplification. Moreover, deletions of the *NFKBIA* and *HLA-A* genes were recurrently captured in 6 (11.5%) and 5 (9.6%) samples. Amplification of *GAB2* and *RPS6KB1* was detected in the baseline plasma of 2 patients (*GAB2* for No. 7 and *RPS6KB1* for No. 16) but not in samples collected thereafter.

Point mutations in breast cancer-related genes were present in 49 of 52 (94.2%) plasma samples and all 18 patients (Supplementary Table S5). Mutations in the hotspot genes *TP53* and *PIK3CA* were recurrently detected in 8 (44.4%) and 7 (27.8%) patients, respectively. Variants in other frequently mutated genes, i.e., *ATM/BRCA2/ERBB2*, were also identified. We also captured rarely documented somatic mutations in the genes *CDK12*, *ROS1* and *TSC2*. CDK12 is a key regulator of transcription and has been correlated with homologous recombination (HR) repair defects in ovarian cancer [24]. A nonsense mutation in *CDK12* (c.3724C>T, p.R1242*) was identified in the baseline and second cycle plasma of patient No. 12.

In summary, somatic genomic alterations in ctDNA including CNVs and point mutations were identified in 50 of 52 (96.2%) blood samples and all 18 patients (100%).

### Serial monitoring of genome alterations in ctDNA

As is always true in administration of anti-HER2 targeted therapy, it's crucial to evaluate the status of *HER2* amplification before initiation of treatment. At baseline we identified *HER2* amplification in only 9 of 18 patients (50.0%) who presented with HER2-positive tumors at diagnosis by histologic review. The status of *HER2* amplification at baseline was not informative because we failed to observe an association between initial ctDNA assay results and the best response achieved. Nevertheless, by comparing the performance of serial ctDNA assays with that of consecutive radiological assessments we found that the dynamics of *HER2* copy number rather than baseline *HER2* amplification status correlated with response to targeted therapy in the real-time management of MBC.

**Figure 1:**
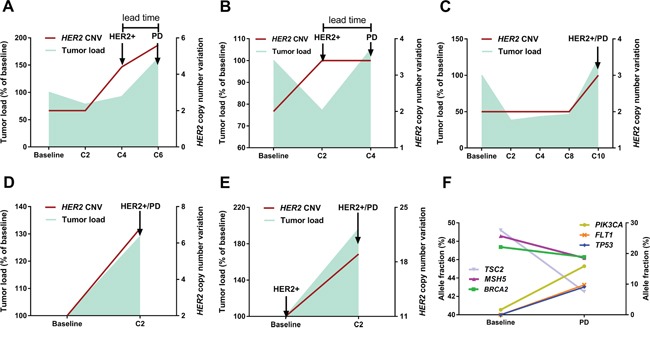
Serial monitoring of genomic alterations in ctDNA (**panel A**, patient No.3) A typical case illustrates the relationship between fluctuation patterns of *HER2* copy number (right Y axis) and dynamics of tumor load (left Y axis). Notably, *HER2* amplification in ctDNA was identified 8 weeks earlier than the clinical establishment of disease progression by CT. (**panel B**, patient No.2) The tumor load moderately decreased after C2 whereas *HER2* copy number was elevated, which was followed by immediate disease progression after C4. (**panel C**, patient No.17; **panel D**, patient No.5; **panel E**, patient No.8) Notable increase in *HER2* copy number and tumor burden was concurrently detected, regardless of *HER2* status at baseline. (**panel F**, patient No.5) Dynamic ctDNA profiling revealed intra-tumor heterogeneity and clonal evolution, as evidenced by the diverging patterns of fluctuation in identified mutations. The left Y axis refers to the allele fractions of mutations in genes *TSC2/MSH5/BRAC2* and the right Y axis to genes *PIK3CA/FLT1/TP53*.

Patient No. 3 is illustrative of the relationship between *HER2* copy number dynamics and outcome (Figure 1A). *HER2* amplified copies were not identified in the ctDNA prior to treatment and remained undetectable after cycle 2 (C2), which coincided with a slight decrease in the tumor load. However, a notable rise in the *HER2* copy number was captured after C4, which further increased until the clinical establishment of disease progression after C6. In other words, monitoring for drug resistance via *HER2* CNV dynamics in ctDNA provided 8 weeks' lead time compared with conventional imaging methods.

The relationship between fluctuation patterns of *HER2* CNV and tumor dynamics was also observed in other cases which were demonstrated in Figure 1 (panel B, C, D, E). For patient No.2 (Figure 1B), the tumor load moderately decreased after C2 whereas *HER2* copy number was elevated in the ctDNA, which was followed by immediate disease progression after C4. This case together with patient No.3 indicated that ctDNA assays might provide early detection of resistance compared with conventional methods. Shown in panels C (patient No.17), D (patient No.5) and E (patient No.8) is the concurrent detection of notable increase in *HER2* copy number and tumor burden, regardless of *HER2* status at baseline.

Moreover, dynamic profiling of somatic mutations in ctDNA identified intra-tumor heterogeneity and resistance-mediating mechanisms. For example, in a patient (No. 5, Figure 1F) diagnosed with multiple liver and bone metastases, a set of gene mutations (*BRCA2*, *MSH5* and *TSC2*) dominated in the baseline plasma while the fraction of *PIK3CA* mutation was low. Subsequent analysis of the plasma collected prior to the establishment of progressive disease revealed diverging patterns in the fractions of mutated genes, with an evident increase in the *PIK3CA* mutation level and moderate decrease in the levels of previously dominant genes. These changes indicated that they derived from different subclones and thus added evidence of intra-tumor heterogeneity. Importantly, we identified much more mutations in the C2 sample than at baseline, with a noticeable rise in the fractions of mutated *TP53* and *FLT1* (Figure 1F), which suggested the increase of clonal heterogeneity at the metastatic sites. Elevation in the level of *PIK3CA* mutation as well as subclonity might account for the observed immediate resistance to targeted therapy.

### ctDNA profiling to identify genomic patterns of resistance

Events of progressive disease occurred in 14 of 18 patients, and primary therapeutic resistance was identified in 6 of them (6/14, 42.9%). For the subset of patients with primary resistance (n=6), the results of pre- and post-treatment ctDNA assays were compared so as to generate clues about the sources of resistance to anti-HER2 therapy.

First and foremost, *HER2* amplification in ctDNA concurred with disease progression in 4 patients (4/6, 66.7%) irrespective of *HER2* status at baseline. However, we failed to capture *HER2* amplification in the ctDNA of the other two patients when a definite increase in the tumor burden was recognized, indicating the role of other resistance-conferring mechanisms. As summarized in Table 1, somatic mutations in gene *TP53* were identified in 3 of 6 patients (50.0%) with primary resistance to anti-HER2 therapy. Other putative mechanisms included variants in genes implicated in the PI3K/Akt/mTOR signaling pathway, i.e. *PIK3CA/PTEN/MTOR* (3/6, 50.0%).

**Table 1:** Genetic alterations in ctDNA associated with resistance to anti-HER2 therapy

Patient ID	Resistance	Gene	Mutation (CDS)	Mutation (Amino Acid)	Oncogenic alteration in COSMIC database
5	Primary	*PIK3CA*	c.[3140A>G]	p.[H1047R]	Yes
		*ERBB2*	c.[3235G>A]	p.[E1079K]	Yes
		*MTOR*	c.[6286G>C]	p.[D2096H]	—
		*ERBB2*	Amplification	—	Yes
		*CDK12*	Amplification	—	Yes
		*TP53*	c.[811G>T]	p.[E271*]	Yes
8	Primary	*ERBB2*	Amplification	—	Yes
		*ROS1*	c.[6316G>A]	p.[A2106T]	Yes
9	Primary	*ATM*	c.[8246A>T]	p.[K2749I]	—
		*TP53*	c.[392A>G]	p.[N131S]	Yes
		*NOTCH1*	c.[4319_4320insC]	p.[I1440fs*?]	—
11	Primary	*ERBB2*	Amplification	—	Yes
		*CDK12*	Amplification	—	Yes
		*MED12*	c.[3745C>A]	p.[L1249I]	—
		*MSH2*	c.[1742T>G]	p.[I581S]	—
12	Primary	*ERBB2*	Amplification	—	Yes
		*TP53*	c.[706T>A]	p.[Y236N]	Yes
14	Primary	*PIK3CA*	c.[1035T>A]	p.[N345K]	Yes
		*CCND1*	Amplification	—	—
		*FGF19*	Amplification	—	—
		*FGF3*	Amplification	—	—
		*FGF4*	Amplification	—	—
		*GPR124*	Amplification	—	—
2	Acquired	*CROT*	c.[1152A>C]	p.[K384N]	—
		*ERBB2*	Amplification	—	Yes
		*CDK12*	Amplification	—	Yes
		*DOT1L*	c.[967G>A]	p.[E323K]	—
3	Acquired	*PIK3CA*	c.[3140A>G]	p.[H1047R]	Yes
		*ERBB2*	Amplification	—	Yes
		*CDK12*	Amplification	—	Yes
		*TP53*	c.[375+2T>G]	—	Yes
4	Acquired	*PIK3CA*	c.[3140A>G]	p.[H1047R]	Yes
		*TP53*	c.[318C>G]	p.[S106R]	Yes
6	Acquired	*RPS14*	c.[218C>A]	p.[A73D]	—
7	Acquired	*ERBB2*	Amplification	—	Yes
		*TP53*	c.[497C>G]	p.[S166*]	Yes
15	Acquired	*MTOR*	c.[1077C>A]	p.[S359R]	—
17	Acquired	*TP53*	c.[672+1G>T]	—	Yes
		*PIK3CA*	c.[1637A>G]	p.[Q546R]	Yes
		*TP53*	c.[833C>G]	p.[P278R]	Yes
		*ERBB2*	Amplification	—	Yes
		*PIK3CA*	c.[3140A>G]	p.[H1047R]	Yes
18	Acquired	*ERBB2*	c.[2264T>C]	p.[L755S]	Yes
		*PTEN*	c.[511C>T]	p.[Q171*]	Yes
		*PIK3CA*	c.[1624G>A]	p.[E542K]	Yes

**Figure 2:**
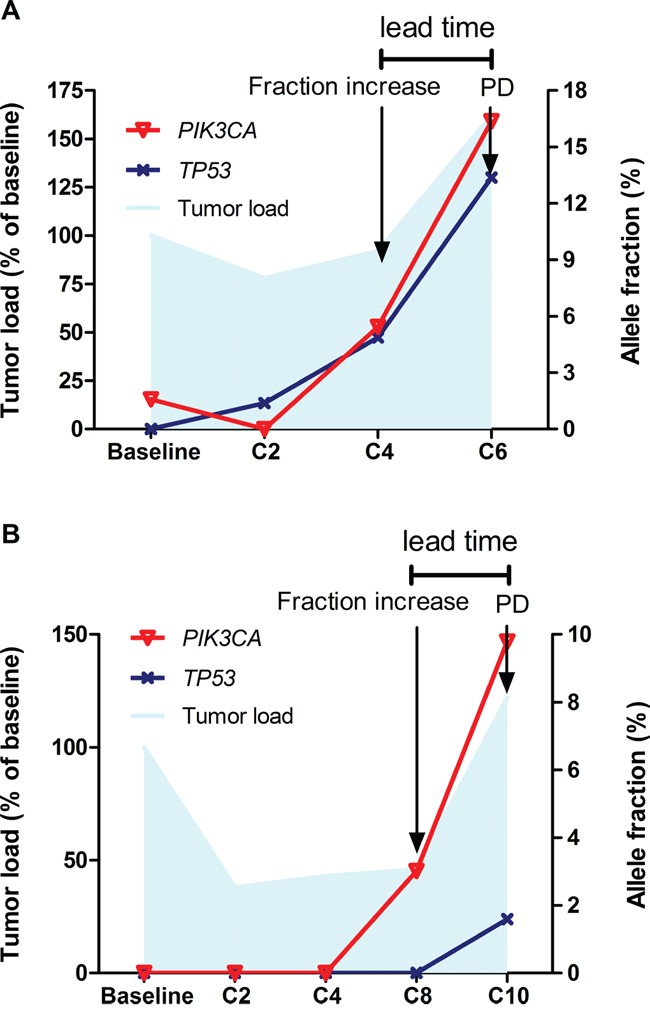
Dynamics of somatic mutations in ctDNA Fluctuations in allele fraction of somatic mutations (right Y axis) generally correlated with tumor burden reflected by imaging method (left Y axis), with increased allele fraction concurring with or even preluding disease progression (**panel A.**, patient No.3; **panel B.**, patient No.17).

With regards to the patients with acquired resistance (N=8), serial ctDNA sequencing has revealed similar findings. *HER2* amplification was identified in 7 of 8 patients. When the tumors responded to the therapy (SD/PR) *HER2* amplified copies were infrequently detected (3/14 samples, 21.4%), whereas at disease progression *HER2* amplification was present in ctDNA in 4 out of 7 samples (4/7, 57.1%). All of the patients had mutations detected. The most frequent mutations identified were *PIK3CA/PTEN/MTOR* (5/8, 62.5%), followed by *TP53* (4/8, 50%). Fluctuations in allele fraction (AF) of dominant mutations generally correlated with tumor burden reflected by imaging method, with increased AF concurring with or even preluding disease progression (Figure 2).

**Figure 3:**
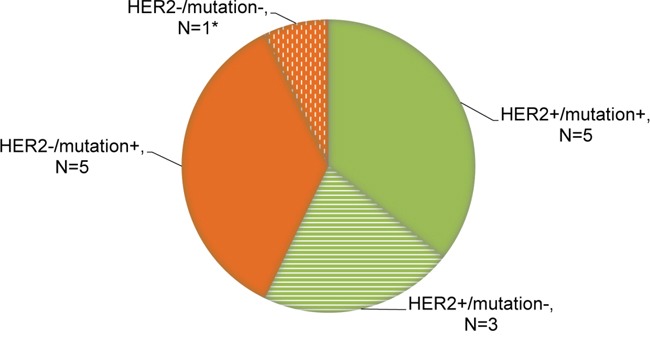
Distribution of genomic patterns of resistance to anti-HER2 therapy Relevant mutations involve genes *TP53/PIK3CA/MTOR/PTEN,* all of which have been identified to correlate with resistance to anti-HER2 therapy. *Patient No. 6 displayed neither *HER2* amplification nor specific mutations but we captured a mutation in *RSP14* prior to progression which might be associated with treatment failure.

Together, based on the dominant markers of resistance described above, we identified 4 patterns of change in ctDNA at the onset of resistance to anti-HER2 therapy as shown by Figure 3. Among the patients with primary or acquired drug resistance (N=14), 5 patients (35.7%) exhibited concurring *HER2* amplification and mutations in genes *TP53/PIK3CA/MTOR/PTEN*. Alterations in these genes have been previously demonstrated to correlate with resistance to HER2 targeted therapeutics. In 8 patients either *HER2* amplification (N=3, 21.4%) or mutations in the genes specified above (N=5, 35.7%) were detected in the ctDNA at disease progression. There was one patient (No. 6) displaying neither *HER2* amplification nor important mutations but we captured a mutation in *RSP14* prior to progression which might be associated with treatment failure.

### Use of ctDNA-based criteria to monitor resistance to targeted therapy

As stated above, ctDNA sequencing enabled the personalized and dynamic profiling of the tumor molecular landscape, which may be exploited to elucidate the tumor genomic response and monitor resistance to targeted therapy. The findings of the present and previous studies indicate that the predictive power of a single-gene-based ctDNA assay is limited in monitoring response to treatment. Here, we proposed a set of combined criteria that could be adopted to identify resistance to HER1/HER2 blockade in HER2-positive MBC. Drug resistance should be indicated during treatment if any of the following situations applies: 1) recurrence or persistence of *HER2* amplification in the blood; 2) emergence or ≥20% increase in the fraction of mutations in any of these resistance-related genes including *TP53/PIK3CA/MTOR/PTEN*. We set the threshold value for the increase of AF as 20% after allowing for the margin of error in ctDNA assay.

Table 2 compares the performance of ctDNA profiling of 34 plasma samples with that of temporally matched CT scans. Specifically, ctDNA analysis utilizing the combined criteria was a highly sensitive approach (sensitivity 85.7%) to detect drug resistance confirmed by CT. However, the relatively low specificity (55.0%) of this method should be interpreted with caution because the discordant evaluations (progression by ctDNA/non-progression by CT, false positivity) were ascribed to the inefficient reflection of tumor response by CT in some cases. After adjusting for this caveat in cases in which ctDNA assay identified resistance earlier than CT (N=6), we obtained a concordance rate up to 82.1%, confirming the robustness of this approach.

**Table 2:** Comparison of ctDNA assay with CT scans to monitor resistance to anti-HER2 therapy

N (%)	CT		Concordance rate (%) ^a^	Adjusted concordance rate (%) ^b^
	Progression n=14	Non-progression n=20		
*HER2* amplification			73.5	80.6
Progression	8 (57.1)	3 (15.0)		
Non-progression	6 (42.9)	17 (85.0)		
Somatic mutations ^c^			58.8	69.0
Progression	8 (57.1)	8 (40.0)		
Non-progression	6 (42.9)	12 (60.0)		
Combined ctDNA criteria			67.6	82.1
Progression	12 (85.7)	9 (45.0)		
Non-progression	2 (14.3)	11 (55.0)		

a Concordance rate=Number of consistent evaluations by ctDNA assay and CT/total number of evaluations×100%

b In some cases, ctDNA assay detected drug resistance earlier than CT did, as confirmed by subsequent imaging assessment. In that situation, the discordant evaluations (progression by ctDNA/non-progression by CT) were ascribed to the inefficient reflection of tumor response by CT and consequently were excluded when calculating the concordance rate.

c In this criterion, relevant mutations involve genes *TP53/PIK3CA/MTOR/PTEN*, all of which have been identified as potential markers of resistance to anti-HER2 therapy.

**Figure 4:**
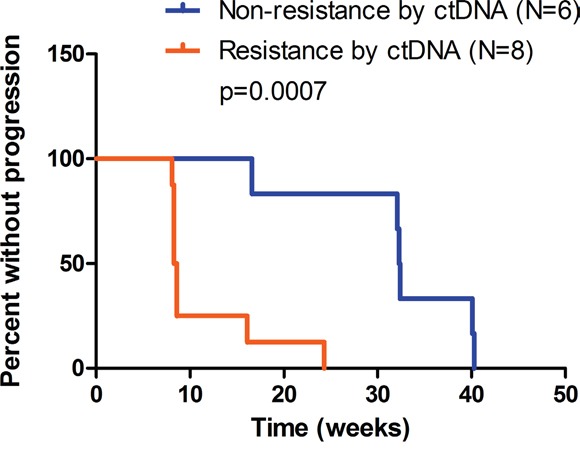
Progression-free survival (PFS) of patients with events of progressive disease (N=14) Based on sequencing data of C2 ctDNA, patients were evaluated as non-resistant (N=6) or resistant (N=8) using pre-defined ctDNA criteria. Non-resistance subset includes patient No.4, 6, 7, 15, 17, 18, and resistance subset includes patient No. 2, 3, 5, 8, 9, 11, 12, 14. Median PFS was 32.4 and 8.5 weeks respectively. P=0.0007 by log-rank test.

Subsequently we sought to explore the prognostic value of ctDNA-based criteria in HER2-positive metastatic disease. Notably, patients with resistance determined by C2 ctDNA results displayed significantly shorter PFS (median 8.5 vs. 32.4 weeks, p=0.0007, Figure 4), suggesting the discriminating role of ctDNA criteria in the prognosis of MBC. The present study is not yet powered to prove that ctDNA-based criteria outperforms Response Evaluation Criteria In Solid Tumors (RECIST), which should be addressed in future study with larger cohorts.

## DISCUSSION

In the present study, we demonstrated that ctDNA genotyping using serially collected samples was an efficient and inherently specific approach to monitor resistance to targeted agent administration and identify emerging mechanisms of resistance in HER2-positive MBC. Compared with previous research, our study is the first to propose ctDNA-based criteria to determine resistance to HER1/HER2 blockade. We also made an initial attempt to investigate the tumor genomic response to anti-HER2 targeted therapy by ctDNA genotyping in the setting of a prospective clinical trial.

For patients with metastatic disease, it's clinically important to precisely determine response and timely detect drug resistance. In this prospective study, we exploited longitudinal analysis of ctDNA to monitor resistance to HER1/HER2 blockade. We found that the dynamics of *HER2* CNVs rather than baseline *HER2* amplification status closely correlated with response to anti-HER2 therapy. Recurrence or persistence of *HER2* amplified copies in the blood heralded resistance-related disease progression.

Nevertheless, the above-described pattern of *HER2* CNV fluctuation was not captured in a considerable fraction of patients (6/14, 42.9%). We adopted target-capture NGS to characterize alterations in a panel of breast cancer-related genes and revealed resistance-related events other than *HER2* CNVs. Putative mechanisms underpinning resistance to anti-HER2 therapy included up-regulation of the PI3K/Akt/mTOR pathway [20], *PTEN* mutation or loss of heterozygosity (LOH) [19, 20], accumulation of the p95 isoform of HER2 [25], signaling from HER family receptors [22] and insulin-like growth factor receptor [26, 27] and activation of estrogen receptor signaling [21]. Here we exploited pre- and post-treatment ctDNA profiling and found that mutations in *TP53* and genes in the PI3K/Akt/mTOR pathway were heavily involved in resistance to HER1/HER2 blockade, adding evidence for the crucial role of these plausible mechanisms. Intriguingly, we also detected a moderate level of *CDK12/ERBB2* co-amplification in resistant cases, but the biological relevance of this observation warrants further exploration.

Given that the agent used in our study is a dual EGFR/HER2 inhibitor, one important issue should be highlighted concerning the predictive value of *EGFR* alteration. Here alteration in gene *EGFR* (amplification) was identified in only one sample (patient No. 3, at disease progression). The very low detection rate of *EGFR* alteration (1/18, 5.6%) has prevented further exploration of its predictive value in our study. Previous findings indicated that *EGFR* mutations were rare if not absent in breast cancer [28, 29] and thus not a suitable predictor for anti-EGFR targeted therapy. Data on *EGFR* amplification rates in breast carcinomas has been inconsistent ranging from 0.8-28% [30–32]. Clinical trials evaluating the efficacy of anti-EGFR therapy in breast cancer have revealed disappointing outcomes [33–35], and correlative studies suggested that EGFR expression/amplification status was not a significant predictor [33, 35]. However, whether limited presence of *EGFR* amplification in ctDNA could predict resistance to dual EGFR/HER2 blockade has yet to be elucidated in future studies.

Our findings have several important implications. First, we demonstrated the clinical utility and validity of serial ctDNA profiling in precise delivery of targeted therapy in HER2-positive MBC. Based on our data, resistance to targeted therapy in HER2-positive MBC should be noted if ctDNA profiling reveals recurrence or persistence of CNVs in *HER2*, or increase in the fraction of certain mutations. These combined criteria exemplify how ctDNA sequencing data can be interpreted to determine drug resistance, which we expect to be useful in the management of various metastatic cancers. Although what degree of change is sufficient to cause shift in clinical management still merits further study, the results derived herein could at least provide hints for rational design of customized ctDNA assays in the future.

Besides, as a multiplex biomarker another purpose of ctDNA sequencing was to identify emerging mechanisms of resistance and propose candidate drug targets for salvage treatment. For example, regarding patients with obvious increase in the fraction of *PI3KCA* mutation before progression, predominant growth of *PIK3CA* mutation-carrying clone probably resulted in treatment failure and PI3K signaling blockade might be the optimal remedy for subsequent management.

Of note, our data convincingly demonstrated the validity of tumor clonal evolution theory. In some cases, we observed discordant patterns of change in somatic genomic mutations, suggesting that these mutations originated from different subclones and the selective pressure of therapeutic intervention finally led to the expansion of resistant clones. Genetic diversification as exemplified by an increase in clonal heterogeneity also promotes the territorial expansion of the tumor. Serial ctDNA analysis substantiated the reiterative and adaptive process of clonal evolution and provided further insights into disease biology.

The present research features several improvements over previous studies. First and foremost, earlier ctDNA studies were mostly retrospective and based on small cohorts, and the patients were treated with a mixture of various chemotherapeutic regimens, which precluded rational inter-study comparisons [36–39]. Conversely, our study was conducted in the setting of a prospective clinical trial, and the study population was uniformly exposed to anti-HER2 targeted therapy. Therefore, the major findings derived from our study would better fit into the practice and guide the optimal administration of HER2 blocking agents in HER2-positive MBC. Second, our study represents an initial attempt to propose a set of ctDNA-based criteria that can be readily used to assess resistance to HER2-targeted therapy during treatment. This actually helped narrow the gap between bench and bedside and further confirmed the clinical validity and utility of ctDNA profiling.

Despite the advantages delineated above, several limitations of our study should be noted. To start with, *HER2* amplification was identified by ctDNA assay in only 13 of 18 patients at any time point, resulting in a relatively low concordance rate with tumor HER2 status (13/18, 72.2%). Although different timing of tissue (primary lesion) and plasma sampling (metastatic setting) may partly account for the disconcordance, possible methodological concerns were still explored. The sensitivity of the ctDNA assay might not suffice to accurately detect copy number variants which could be addressed in future study by improving the coverage depth of sequencing. This poor concordance could also be attributed to other technical issues such as inadequate plasma available for assay and high background levels of circulating wild-type DNA. Secondly, the threshold value for the degree of increase in the fraction of mutations was laid down at 20% so that optimal sensitivity/specificity of the ctDNA-based criteria could be derived, in a somewhat arbitrary way. The level of alteration in somatic mutations sufficient to initiate a change in clinical management needs to be specified in large-scale prospective trials. Thirdly, a comparison of genomic data from non-treated HER2-positive breast cancer patients is lacking, to be able to get a perspective of the therapy effect in driving HER2 CNVs and vice versa. Moreover, the small number of evaluable patients prevented rigorous statistical analysis, so the present study was less powered to arrive at statistically sound conclusions. Our findings derived from this proof-of-principle study, which represent a logical evolution in the field of ctDNA, warrant further validation in larger series of patients.

## MATERIALS AND METHODS

### Patients and sample collection

Blood samples were prospectively collected from patients with HER2-positive MBC who participated in a clinical trial (NCT01937689) evaluating the safety and efficacy of pyrotinib, an oral anti-HER1/HER2 tyrosine kinase inhibitor, which was administered daily on a 28 d/cycle regimen [23]. All enrolled patients provided written informed consent for serial blood collection and sample assay. The study was approved by the institutional review board of Cancer Hospital, Chinese Academy of Medical Sciences. Blood samples were collected before the initiation of treatment and after every two cycles of therapy until disease progression. Plasma was separated by centrifugation at 1,600 g for 10 min at 4°C and the supernatant was then centrifuged for a second time at 16,000g for 10 min at 4°C to remove the cellular components. Both plasma and peripheral blood cells were aliquoted and stored at −80 °C until ctDNA and genomic DNA (gDNA) extraction. In order to prevent lysis of white blood cell and thus preserve the integrity of ctDNA, blood samples were processed and frozen within 2 hours of sample collection.

### DNA extraction

ctDNA and gDNA were extracted using the QIAamp Circulating Nucleic Acid Kit (Qiagen) and QIAamp DNA Blood Mini Kit (Qiagen) respectively, according to the manufacturer's instructions. ctDNA was sequenced to detect somatic alterations, whereas gDNA was adopted as normal control.

### Target capture and next-generation sequencing

A total of 368 genes were selected from four sources: 1) known oncogenes and tumor suppressor genes; 2) genes that are targets of agents approved by the FDA or have been assessed in clinical trials; 3) genes implicated in major cancer-related signaling pathways; 4) genes identified in the findings of the TCGA network which covers 12 cancer types. The target-capture region was 1.9 Mb in size and designed for all exons from 368 genes. Sequencing libraries were prepared from ctDNA using KAPA DNA Library Preparation Kits (Kapa Biosystems, Inc.), and gDNA sequencing libraries were prepared using the protocols recommended by the Illumina TruSeq DNA Library Preparation Kit. For samples close to the minimum input requirement, additional pre-capture PCR cycles were performed to generate sufficient PCR product for hybridization. The libraries were hybridized to the 1.9-Mb custom-designed probes (NimbleGen, Roche) of biotinylated oligonucleotides. DNA sequencing was performed on a HiSeq2500 sequencing system (Illumina, San Diego, CA) with 2*101 bp paired-end reads. The reads were aligned to the human genome build GRCh37 using BWA (a Burrows–Wheeler aligner). Somatic single nucleotide variant (sSNV) and indel calls were generated using MuTect and GATK, respectively. Somatic copy number alterations were identified with CONTRA (COpy Number Targeted Resequencing Analysis, Supplementary Figure S1).

### Sequencing data analysis

All variants identified by the bioinformatics pipeline were manually reviewed by an experienced bioinformatics director to assess the quality of base calls, the mapping quality of the reads, and the overall read depth at the site. Variations meeting any of the criteria listed below were filtered: low base quality (Phred score <13) in all reads supporting the variation; mutant reads all in the plus or minus strand; all the reads with mutant allele did not meet mapping confidently (quality score >=30); reads support at variant position <3; and variants detected near the start/end of sequencing reads. For a given variant in plasma ctDNA, allele fraction = sequencing read count of alternate alleles / (sequencing read count of reference alleles + sequencing read count of alternate alleles) *100%.

### Statistical analysis

Tumor burden was measured as the sum of the largest diameters of the target lesions. Clinical response was evaluated every two cycles as per RECIST v1.1 [40]. Primary resistance referred to disease progression on the first restaging CT scan, while acquired resistance was defined as progression after initial response to HER2 targeted therapy. PFS was defined as the interval between the initiation of treatment and the date of disease progression or death from any cause. Cases without progression or death events were censored at the date of last follow-up. Survival curves were estimated using the Kaplan–Meier method and unadjusted comparison of these estimates was performed using log-rank test. We assessed the clinical utility of ctDNA assay in monitoring drug resistance in terms of sensitivity, specificity and concordance rate. Using temporally paired plasma samples and CT scans, the concordance rate was defined as the number of consistent evaluations by ctDNA assay and CT out of the total number of evaluations (N=34). Sensitivity was calculated as the proportion of progression events decided by both ctDNA assay and CT scans among all the progression events established by CT (N=14). Specificity referred to the percentage of non-progression evaluations determined by both ctDNA assay and CT scans in total non-progression evaluations assessed by CT (N=20). All reported p values were two-sided, and p<0.05 was considered statistically significant. All statistical analyses were performed using SPSS version 19.0 (SPSS Company, Chicago, IL).

## SUPPLEMENTARY MATERIALS FIGURE AND TABLES




