# Supplementary material for: ctDNA dynamics: a novel indicator to track resistance in metastatic breast cancer treated with anti-HER2 therapy
Source: Oncotarget. 2016 Sep 1;7(40):66020–31. doi: 10.18632/oncotarget.11791 (PMC5323211; doi:10.18632/oncotarget.11791)
Supplement: Supplementary file 1 [file oncotarget-07-66020-s001.pdf]

ctDNA dynamics: a novel indicator to track resistance in metastatic breast cancer treated with anti-HER2 therapy

SUPPLEMENTARY FIGURE AND TABLES

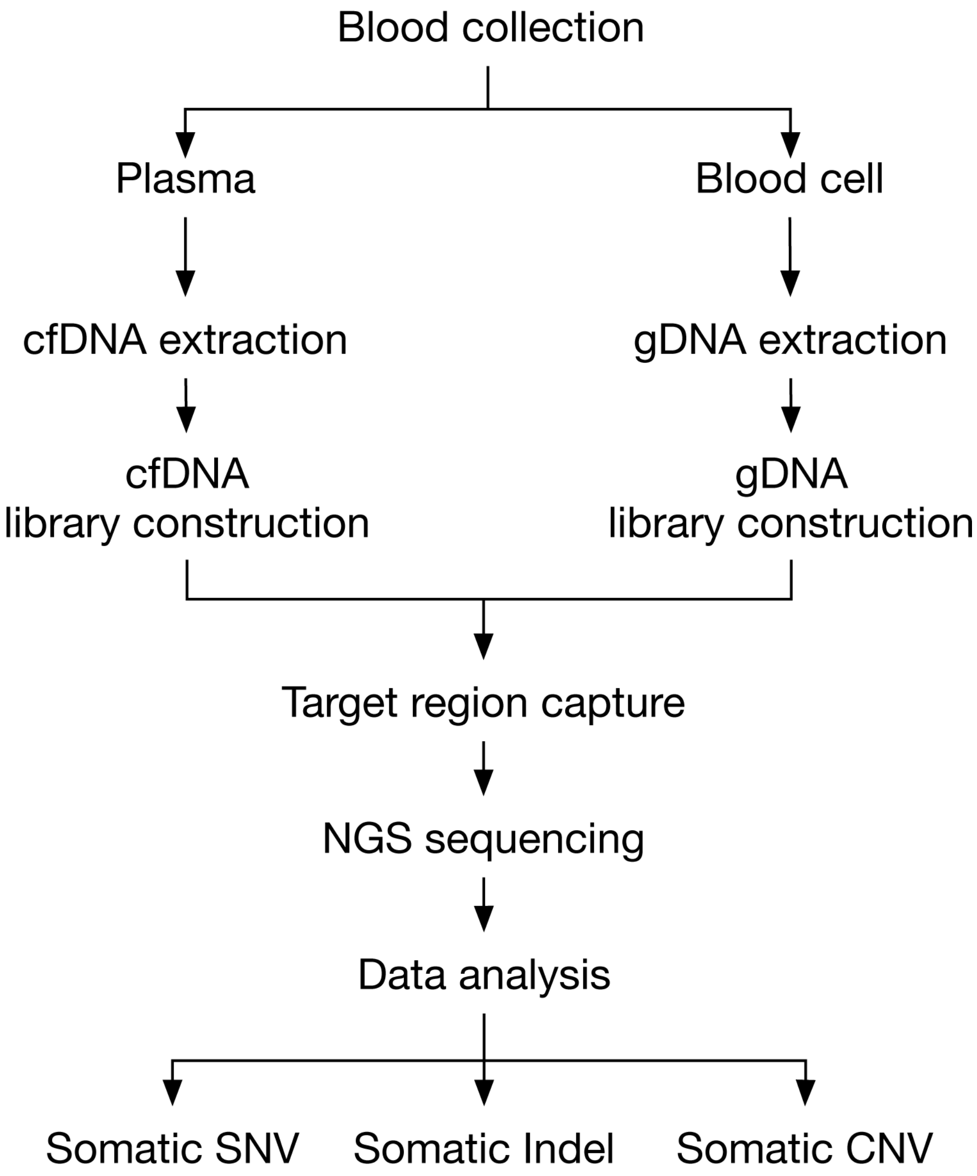

Supplementary Figure S1: Workflow of sample assay.

Supplementary Table S1: Clinical characteristics of study population

See Supplementary File 1

Supplementary Table S2: List of target region genes

See Supplementary File 1

Supplementary Table S3: Detailed sequencing data for 18 patients

See Supplementary File 1

Supplementary Table S4: Somatic copy number variants (CNVs) identified in 52 plasma samples

See Supplementary File 1

Supplementary Table S5: Non-silent SNV and small indels identified in 52 plasma samples

See Supplementary File 1
