# Supplementary material for: ctDNA dynamics: a novel indicator to track resistance in metastatic breast cancer treated with anti-HER2 therapy
Source: Oncotarget. 2016 Sep 1;7(40):66020–31. doi: 10.18632/oncotarget.11791 (PMC5323211; doi:10.18632/oncotarget.11791)
Supplement: Supplementary file 2 [file oncotarget-07-66020-s002.doc]

**Summary of supplementary tables**

Supplementary Table 1. Clinical characteristics of study population

Supplementary Table 2. List of target region genes

Supplementary Table 3. Detailed sequencing data for 18 patients

Supplementary Table 4. Somatic copy number variants (CNVs) identified in 52 plasma samples

Supplementary Table 5. Non-silent SNV and small indels identified in 52 plasma samples

Supplementary Table 1. Clinical characteristics of study population

| **Patient ID** | **Age** | **ECOG** | **Histology** | **Grade** | **ER** | **PgR** | **Visceral involvement** | **Cycles of therapy** | **Best response achieved** | **Disease progression** | **PFS (week)** | **Plasma samples tested** |
| --- | --- | --- | --- | --- | --- | --- | --- | --- | --- | --- | --- | --- |
| **1** | 54 | 0 | IDC | II | Positive | Positive | Yes | 18 | PR | No | 72.1 | 2 |
| **2** | 32 | 0 | IDC | III | Negative | Negative | No | 4 | SD | Yes | 16.1 | 3 |
| **3** | 32 | 0 | IDC | II | Negative | Negative | Yes | 6 | SD | Yes | 24.3 | 4 |
| **4** | 48 | 0 | IDC | III | Positive | Negative | No | 8 | PR | Yes | 32.4 | 4 |
| **5** | 38 | 0 | IDC | II | Positive | Positive | Yes | 2 | PD | Yes | 8.3 | 2 |
| **6** | 50 | 0 | DCIS with microinvasive carcinoma | NA | Negative | Positive | Yes | 8 | PR | Yes | 32.3 | 4 |
| **7** | 61 | 0 | IDC | II | Negative | Positive | Yes | 4 | SD | Yes | 16.6 | 3 |
| **8** | 50 | 0 | IDC | III | Negative | Negative | Yes | 2 | PD | Yes | 8.6 | 2 |
| **9** | 41 | 0 | IDC | II | Positive | Positive | No | 2 | PD | Yes | 8.1 | 2 |
| **10** | 45 | 0 | IDC | III | Negative | Negative | Yes | 16 | SD | No | 64.3 | 2 |
| **11** | 29 | 0 | IDC | II | Negative | Positive | Yes | 2 | PD | Yes | 8.3 | 2 |
| **12** | 51 | 0 | IDC | II | Negative | Negative | No | 2 | PD | Yes | 8.6 | 2 |
| **13** | 44 | 0 | IDC | III | Positive | Positive | Yes | 2 | SD | No | 8.1 | 2 |
| **14** | 30 | 0 | IDC | II | Positive | Positive | Yes | 2 | PD | Yes | 8.3 | 2 |
| **15** | 55 | 0 | IDC | II | Positive | Positive | No | 8 | PR | Yes | 32.1 | 4 |
| **16** | 49 | 0 | IDC | II | Positive | Positive | Yes | 15 | PR | No | 60.4 | 2 |
| **17** | 42 | 0 | IDC | II | Positive | Positive | Yes | 10 | PR | Yes | 40.1 | 5 |
| **18** | 50 | 0 | IDC&ILC | II | Positive | Positive | No | 10 | PR | Yes | 40.3 | 5 |

**Abbreviations: DCIS ductal carcinoma in situ, ER estrogen receptor, IDC invasive ductal carcinoma, ILC invasive lobular carcinoma, NA not applicable, PD progressional disease, PFS progression-free survival, PgR progesterone receptor, PR partial response, SD stable disease**

**Grey color indicates the characteristics of disease at primary diagnosis.**

Supplementary Table 2. List of target region genes

| **1. known oncogenes and tumor suppressor genes (TSG)** | | | | | | | | | | |
| --- | --- | --- | --- | --- | --- | --- | --- | --- | --- | --- |
| **Gene Symbol** | | | **Gene Name** | | | | | **Classification** | | |
| *ABL1* | | | c-abl oncogene 1, receptor tyrosine kinase | | | | | Oncogene | | |
| *ACVR1B* | | | activin A receptor, type IB | | | | | TSG | | |
| *AKT1* | | | v-akt murine thymoma viral oncogene homolog 1 | | | | | Oncogene | | |
| *ALK* | | | anaplastic lymphoma receptor tyrosine kinase | | | | | Oncogene | | |
| *APC* | | | adenomatous polyposis coli | | | | | TSG | | |
| *AR* | | | androgen receptor | | | | | Oncogene | | |
| *ARID1A* | | | AT rich interactive domain 1A (SWI-like) | | | | | TSG | | |
| *ARID1B* | | | AT rich interactive domain 1B (SWI1-like) | | | | | TSG | | |
| *ARID2* | | | AT rich interactive domain 2 (ARID, RFX-like) | | | | | TSG | | |
| *ASXL1* | | | additional sex combs like 1 (Drosophila) | | | | | TSG | | |
| *ATM* | | | similar to Serine-protein kinase ATM (Ataxia telangiectasia mutated) (A-T, mutated); ataxia telangiectasia mutated | | | | | TSG | | |
| *ATRX* | | | alpha thalassemia/mental retardation syndrome X-linked (RAD54 homolog, S. cerevisiae) | | | | | TSG | | |
| *AXIN1* | | | axin 1 | | | | | TSG | | |
| *B2M* | | | beta-2-microglobulin | | | | | TSG | | |
| *BAP1* | | | BRCA1 associated protein-1 (ubiquitin carboxy-terminal hydrolase) | | | | | TSG | | |
| *BCL2* | | | B-cell CLL/lymphoma 2 | | | | | Oncogene | | |
| *BCOR* | | | BCL6 co-repressor | | | | | TSG | | |
| *BRAF* | | | v-raf murine sarcoma viral oncogene homolog B1 | | | | | Oncogene | | |
| *BRCA1* | | | breast cancer 1, early onset | | | | | TSG | | |
| *BRCA2* | | | breast cancer 2, early onset | | | | | TSG | | |
| *CARD11* | | | caspase recruitment domain family, member 11 | | | | | Oncogene | | |
| *CASP8* | | | caspase 8, apoptosis-related cysteine peptidase | | | | | TSG | | |
| *CBL* | | | Cas-Br-M (murine) ecotropic retroviral transforming sequence | | | | | Oncogene | | |
| *CDC73* | | | cell division cycle 73, Paf1/RNA polymerase II complex component, homolog (S. cerevisiae) | | | | | TSG | | |
| *CDH1* | | | cadherin 1, type 1, E-cadherin (epithelial) | | | | | TSG | | |
| *CDKN2A* | | | cyclin-dependent kinase inhibitor 2A (melanoma, p16, inhibits CDK4) | | | | | TSG | | |
| *CEBPA* | | | CCAAT/enhancer binding protein (C/EBP), alpha | | | | | TSG | | |
| *CIC* | | | capicua homolog (Drosophila) | | | | | TSG | | |
| *CREBBP* | | | CREB binding protein | | | | | TSG | | |
| *CRLF2* | | | cytokine receptor-like factor 2 | | | | | Oncogene | | |
| *CSF1R* | | | colony stimulating factor 1 receptor | | | | | Oncogene | | |
| *CTNNB1* | | | catenin (cadherin-associated protein), beta 1, 88kDa | | | | | Oncogene | | |
| *CYLD* | | | cylindromatosis (turban tumor syndrome) | | | | | TSG | | |
| *DAXX* | | | death-domain associated protein | | | | | TSG | | |
| *DNMT1* | | | DNA (cytosine-5-)-methyltransferase 1 | | | | | Oncogene | | |
| *DNMT3A* | | | DNA (cytosine-5-)-methyltransferase 3 alpha | | | | | Oncogene | | |
| *EGFR* | | | epidermal growth factor receptor (erythroblastic leukemia viral (v-erb-b) oncogene homolog, avian) | | | | | Oncogene | | |
| *EP300* | | | E1A binding protein p300 | | | | | TSG | | |
| *ERBB2* | | | v-erb-b2 erythroblastic leukemia viral oncogene homolog 2, neuro/glioblastoma derived oncogene homolog (avian) | | | | | Oncogene | | |
| *EZH2* | | | enhancer of zeste homolog 2 (Drosophila) | | | | | Oncogene | | |
| *FAM123B* | | | family with sequence similarity 123B | | | | | TSG | | |
| *FBXW7* | | | F-box and WD repeat domain containing 7 | | | | | TSG | | |
| *FGFR2* | | | fibroblast growth factor receptor 2 | | | | | Oncogene | | |
| *FGFR3* | | | fibroblast growth factor receptor 3 | | | | | Oncogene | | |
| *FLT3* | | | fms-related tyrosine kinase 3 | | | | | Oncogene | | |
| *FOXL2* | | | forkhead box L2 | | | | | Oncogene | | |
| *FUBP1* | | | far upstream element (FUSE) binding protein 1 | | | | | TSG | | |
| *GATA1* | | | GATA binding protein 1 (globin transcription factor 1) | | | | | TSG | | |
| *GATA2* | | | GATA binding protein 2 | | | | | Oncogene | | |
| *GATA3* | | | GATA binding protein 3 | | | | | TSG | | |
| *GNA11* | | | guanine nucleotide binding protein (G protein), alpha 11 (Gq class) | | | | | Oncogene | | |
| *GNAQ* | | | guanine nucleotide binding protein (G protein), q polypeptide | | | | | Oncogene | | |
| *GNAS* | | | GNAS complex locus | | | | | Oncogene | | |
| *H3F3A* | | | H3 histone, family 3B (H3.3B); H3 histone, family 3A pseudogene; H3 histone, family 3A; similar to H3 histone, family 3B; similar to histone H3.3B | | | | | Oncogene | | |
| *HIST1H3B* | | | histone cluster 1, H3j; histone cluster 1, H3i; histone cluster 1, H3h; histone cluster 1, H3g; histone cluster 1, H3f; histone cluster 1, H3e; histone cluster 1, H3d; histone cluster 1, H3c; histone cluster 1, H3b; histone cluster 1, H3a; histone cluster 1, H2ad; histone cluster 2, H3a; histone cluster 2, H3c; histone cluster 2, H3d | | | | | Oncogene | | |
| *HNF1A* | | | HNF1 homeobox A | | | | | TSG | | |
| *HRAS* | | | v-Ha-ras Harvey rat sarcoma viral oncogene homolog | | | | | Oncogene | | |
| *IDH1* | | | isocitrate dehydrogenase 1 (NADP+), soluble | | | | | Oncogene | | |
| *IDH2* | | | isocitrate dehydrogenase 2 (NADP+), mitochondrial | | | | | Oncogene | | |
| *JAK1* | | | Janus kinase 1 | | | | | Oncogene | | |
| *JAK2* | | | Janus kinase 2 | | | | | Oncogene | | |
| *JAK3* | | | Janus kinase 3 | | | | | Oncogene | | |
| *KDM5C* | | | lysine (K)-specific demethylase 5C | | | | | TSG | | |
| *KDM6A* | | | lysine (K)-specific demethylase 6A | | | | | TSG | | |
| *KIT* | | | similar to Mast/stem cell growth factor receptor precursor (SCFR) (Proto-oncogene tyrosine-protein kinase Kit) (c-kit) (CD117 antigen); v-kit Hardy-Zuckerman 4 feline sarcoma viral oncogene homolog | | | | | Oncogene | | |
| *KLF4* | | | Kruppel-like factor 4 | | | | | Oncogene | | |
| *KRAS* | | | v-Ki-ras2 Kirsten rat sarcoma viral oncogene homolog | | | | | Oncogene | | |
| *MAP2K1* | | | mitogen-activated protein kinase kinase 1 | | | | | Oncogene | | |
| *MAP3K1* | | | mitogen-activated protein kinase kinase kinase 1 | | | | | TSG | | |
| *MED12* | | | mediator complex subunit 12 | | | | | Oncogene | | |
| *MEN1* | | | multiple endocrine neoplasia I | | | | | TSG | | |
| *MET* | | | met proto-oncogene (hepatocyte growth factor receptor) | | | | | Oncogene | | |
| *MLH1* | | | mutL homolog 1, colon cancer, nonpolyposis type 2 (E. coli) | | | | | TSG | | |
| *MLL2* | | | myeloid/lymphoid or mixed-lineage leukemia 2 | | | | | TSG | | |
| *MLL3* | | | myeloid/lymphoid or mixed-lineage leukemia 3 | | | | | TSG | | |
| *MPL* | | | myeloproliferative leukemia virus oncogene | | | | | Oncogene | | |
| *MSH2* | | | mutS homolog 2, colon cancer, nonpolyposis type 1 (E. coli) | | | | | TSG | | |
| *MSH6* | | | mutS homolog 6 (E. coli) | | | | | TSG | | |
| *MYD88* | | | myeloid differentiation primary response gene (88) | | | | | Oncogene | | |
| *NCOR1* | | | nuclear receptor co-repressor 1 | | | | | TSG | | |
| *NF1* | | | neurofibromin 1 | | | | | TSG | | |
| *NF2* | | | neurofibromin 2 (merlin) | | | | | TSG | | |
| *NFE2L2* | | | nuclear factor (erythroid-derived 2)-like 2 | | | | | Oncogene | | |
| *NOTCH1* | | | Notch homolog 1, translocation-associated (Drosophila) | | | | | TSG | | |
| *NOTCH2* | | | Notch homolog 2 (Drosophila) | | | | | TSG | | |
| *NPM1* | | | nucleophosmin 1 (nucleolar phosphoprotein B23, numatrin) pseudogene 21; hypothetical LOC100131044; similar to nucleophosmin 1; nucleophosmin (nucleolar phosphoprotein B23, numatrin) | | | | | TSG | | |
| *NRAS* | | | neuroblastoma RAS viral (v-ras) oncogene homolog | | | | | Oncogene | | |
| *PAX5* | | | paired box 5 | | | | | TSG | | |
| *PBRM1* | | | polybromo 1 | | | | | TSG | | |
| *PDGFRA* | | | platelet-derived growth factor receptor, alpha polypeptide | | | | | Oncogene | | |
| *PHF6* | | | PHD finger protein 6 | | | | | TSG | | |
| *PIK3CA* | | | phosphoinositide-3-kinase, catalytic, alpha polypeptide | | | | | Oncogene | | |
| *PIK3R1* | | | phosphoinositide-3-kinase, regulatory subunit 1 (alpha) | | | | | TSG | | |
| *PPP2R1A* | | | protein phosphatase 2 (formerly 2A), regulatory subunit A, alpha isoform | | | | | Oncogene | | |
| *PRDM1* | | | PR domain containing 1, with ZNF domain | | | | | TSG | | |
| *PTCH1* | | | patched homolog 1 (Drosophila) | | | | | TSG | | |
| *PTEN* | | | phosphatase and tensin homolog; phosphatase and tensin homolog pseudogene 1 | | | | | TSG | | |
| *PTPN11* | | | protein tyrosine phosphatase, non-receptor type 11; similar to protein tyrosine phosphatase, non-receptor type 11 | | | | | Oncogene | | |
| *RB1* | | | retinoblastoma 1 | | | | | TSG | | |
| *RET* | | | ret proto-oncogene | | | | | Oncogene | | |
| *RNF43* | | | ring finger protein 43 | | | | | TSG | | |
| *RUNX1* | | | runt-related transcription factor 1 | | | | | TSG | | |
| *SETBP1* | | | SET binding protein 1 | | | | | Oncogene | | |
| *SETD2* | | | SET domain containing 2 | | | | | TSG | | |
| *SF3B1* | | | splicing factor 3b, subunit 1, 155kDa | | | | | Oncogene | | |
| *SMAD2* | | | SMAD family member 2 | | | | | TSG | | |
| *SMAD4* | | | SMAD family member 4 | | | | | TSG | | |
| *SMARCA4* | | | SWI/SNF related, matrix associated, actin dependent regulator of chromatin, subfamily a, member 4 | | | | | TSG | | |
| *SMARCB1* | | | SWI/SNF related, matrix associated, actin dependent regulator of chromatin, subfamily b, member 1 | | | | | TSG | | |
| *SMO* | | | smoothened homolog (Drosophila) | | | | | Oncogene | | |
| *SOCS1* | | | suppressor of cytokine signaling 1 | | | | | TSG | | |
| *SOX9* | | | SRY (sex determining region Y)-box 9 | | | | | TSG | | |
| *SPOP* | | | speckle-type POZ protein | | | | | Oncogene | | |
| *SRSF2* | | | SRSF2 serine/arginine-rich splicing factor 2 | | | | | Oncogene | | |
| *STAG2* | | | stromal antigen 2 | | | | | TSG | | |
| *STK11* | | | serine/threonine kinase 11 | | | | | TSG | | |
| *TET2* | | | tet oncogene family member 2 | | | | | TSG | | |
| *TNFAIP3* | | | tumor necrosis factor, alpha-induced protein 3 | | | | | TSG | | |
| *TP53* | | | tumor protein p53 | | | | | TSG | | |
| *TRAF7* | | | TNF receptor-associated factor 7 | | | | | TSG | | |
| *TSC1* | | | tuberous sclerosis 1 | | | | | TSG | | |
| *TSHR* | | | thyroid stimulating hormone receptor | | | | | Oncogene | | |
| *U2AF1* | | | U2 small nuclear RNA auxiliary factor 1 | | | | | Oncogene | | |
| *VHL* | | | von Hippel-Lindau tumor suppressor | | | | | TSG | | |
| *WT1* | | | Wilms tumor 1 | | | | | TSG | | |
| **2. genes that are targets of agents approved by FDA or assessed in clinical trials** | | | | | | | | | | |
| **Gene** | | **Types of recurrent alterations** | | | | **Therapeutic Agents** | | | | |
| *AKT1* | | Mutation | | | | mTOR inhibitors | | | | |
| *AKT2* | | Mutation; Amplification | | | | mTOR inhibitors | | | | |
| *AKT3* | | Rearrangement; Mutation | | | | mTOR inhibitors | | | | |
| *BRAF* | | Mutation; Amplification; Rearrangement | | | | Vemurafenib, Dabrafenib, RAF inhibitors,MEK inhibitors | | | | |
| *BRCA1* | | Inactivation | | | | PARP Inhibitor | | | | |
| *BRCA2* | | Inactivation | | | | PARP Inhibitor | | | | |
| *EGFR* | | Mutation; Amplification; Rearrangement | | | | Erlotinib, Gefitinib, Afatinib,EGFR Inhibitors | | | | |
| *ERBB2* | | Amplification; Mutations | | | | Trastuzumab, Lapatinib, TDM1, Pertuzumab | | | | |
| *ESR1* | | Amplification; Mutations | | | | Hormonal therapy | | | | |
| *GNA11* | | Mutation | | | | MAPK pathway inhibitors | | | | |
| *GNAQ* | | Mutation | | | | MAPK pathway inhibitors | | | | |
| *IDH1* | | Mutation | | | | IDH inhibitorss | | | | |
| *IDH2* | | Mutation | | | | IDH inhibitorss | | | | |
| *JAK2* | | Mutation | | | | ruxolitinib, JAK inhibitors | | | | |
| *JAK3* | | Mutation | | | | tofacitinib, JAK inhibitors | | | | |
| *KIT* | | Mutation; Amplification | | | | Imatinib, Sunitinib,KIT Inhibitors | | | | |
| *KRAS* | | Mutation | | | | Cetuximab,MEK inhibitors | | | | |
| *MET* | | Amplification | | | | Gefitinib, Erlotinib,Afatinib,EGFR inhibitors,Crizotinib, MET inhibitors | | | | |
| *MTOR* | | Mutation | | | | mTOR inhibitors | | | | |
| *NRAS* | | Mutation | | | | Vemurafenib, Dabrafenib, RAF inhibitors,MEK inhibitors | | | | |
| *PDGFRA* | | Mutation; Rearrangement | | | | Imatininb | | | | |
| *PDGFRB* | | Rearrangement | | | | Imatininb | | | | |
| *PIK3CA* | | Mutations | | | | PI3K/AKT/mTOR inhibitors | | | | |
| *PTCH1* | | Mutations | | | | Vismodegib, hedgehog inhibitors | | | | |
| *PTEN* | | Inactivation | | | | PI3K/AKT/mTOR inhibitors,PARP inhinitors | | | | |
| *RB1* | | Inactivation | | | | CDK inhibitors | | | | |
| *RET* | | Mutation; Rearrangement | | | | Sorafenib, vandetinib, RET Inhibitors | | | | |
| *SMO* | | Mutations | | | | Vismodegib, hedgehog inhibitors | | | | |
| *STK11* | | Inactivation | | | | mTOR inhibitors,Dasatinib, src inhibitors,FAK inhibitors | | | | |
| *TSC1* | | Inactivation | | | | mTOR inhibitors | | | | |
| *TSC2* | | Inactivation | | | | mTOR inhibitors | | | | |
| *ABL1* | | Rearrangement; Mutation | | | | Imatinib, Dasatinib, Nilotinib | | | | |
| *ALK* | | Rearrangement; Mutation; Amplification | | | | Crizotinib, ALK inhibitors | | | | |
| *APC* | | Inactivation | | | | WNT inhibitors | | | | |
| *AR* | | Amplification、mutation | | | | Androgen Deprivation,Enzalutamide、Flutamide | | | | |
| *ARAF* | | Mutation | | | | Sorafenib, Vemurafenib, Dabrafenib, RAF inhibitors | | | | |
| *ATM* | | Inactivation | | | | PARP Inhibitors | | | | |
| *ATR* | | Inactivation | | | | PARP Inhibitors | | | | |
| *AURKA* | | Amplification | | | | AURKA Inhibitors | | | | |
| *BAP1* | | Inactivation | | | | HDAC Inhibitors | | | | |
| *BCL2* | | Rearrangement | | | | BCL2 inhibitors | | | | |
| *BRD2* | | Rearrangement | | | | HDAC Inhibitors,Bromodomain inhibitors | | | | |
| *BRD3* | | Rearrangement | | | | HDAC Inhibitors,Bromodomain inhibitors | | | | |
| *BRD4* | | Rearrangement | | | | HDAC Inhibitors,Bromodomain inhibitors | | | | |
| *NUTM1* | | Rearrangement | | | | HDAC Inhibitors,Bromodomain inhibitors | | | | |
| *CCND1* | | Amplification; Rearrangement | | | | Hormone therapy,CDK4/6 inhibitors,Pazopanib,FGFR inhibitor | | | | |
| *CCND2* | | Amplification; Rearrangement | | | | CDK4/6 inhibitors,Pazopanib,FGFR inhibitor | | | | |
| *CCND3* | | Amplification; Rearrangement | | | | CDK4/6 inhibitors,Pazopanib,FGFR inhibitor | | | | |
| *CCNE1* | | Amplification | | | | CKD2 inhibitor | | | | |
| *CDK4* | | Amplification; Mutation | | | | CDK4/6 inhibitors | | | | |
| *CDK6* | | Amplification; Rearrangement | | | | CDK4/6 inhibitors | | | | |
| *CDKN1A* | | Inactivation | | | | CDK inhibitors | | | | |
| *CDKN1B* | | Inactivation | | | | CDK inhibitors | | | | |
| *CDKN2A* | | Inactivation | | | | CDK4/6 inhibitors | | | | |
| *CDKN2B* | | Inactivation | | | | CDK4/6 inhibitors | | | | |
| *CRKL* | | Amplification | | | | Gefitinib, Erlotinib, Afatinib,EGFR inhibitors,Vemurafenib, Dabrafenib, RAF inhibitors,Dasatinib,SRC inhibitors | | | | |
| *CTNNB1* | | Mutations | | | | WNT inhibitors | | | | |
| *DDR2* | | Mutations | | | | Dasatinib | | | | |
| *DNMT3A* | | Mutations | | | | DNAMT inhibitors | | | | |
| *EPHA3* | | Mutation; Amplification | | | | Dasatinib,Ephrin inhibitors | | | | |
| *ERBB3* | | Mutations | | | | Pertuzumab | | | | |
| *ERBB4* | | Mutation | | | | Lapatinib | | | | |
| *ERG* | | Rearrangement | | | | PARP Inhibitor | | | | |
| *EZH2* | | Mutations | | | | EZH2 inhibitors | | | | |
| *FBXW7* | | Inactivation | | | | mTOR inhibitors,Tubulins | | | | |
| *FGFR1* | | Amplification | | | | FGFR Inhibitors | | | | |
| *FGFR2* | | Mutations; Rearrangement | | | | FGFR Inhibitors | | | | |
| *FGFR3* | | Mutations; Rearrangement | | | | FGFR Inhibitors，Carfilzomib | | | | |
| *FLCN* | | Inactivation | | | | Everolimus, Temsirolimus, mTOR inhibitors | | | | |
| *FLT3* | | Mutation | | | | Sunitinib, FLT3 inhibitors | | | | |
| *GNAS* | | Mutation | | | | JAK inhibitors | | | | |
| *HRAS* | | Mutation | | | | MAPK pathway inhibitors | | | | |
| *IGF1R* | | Amplification | | | | IFG1-R Inhibitor | | | | |
| *KDR* | | Mutations | | | | KDR inhibitors | | | | |
| *MAP2K1* | | Mutation | | | | Vemurafenib, MEK inhibitors | | | | |
| *MAP2K2* | | Mutations | | | | MEK inhibitors | | | | |
| *MAPK1* | | Amplification | | | | Erlotinib, Gefitinib, EGFR Inhibitors | | | | |
| *MAPK3* | | Amplification | | | | Erlotinib, Gefitinib, EGFR Inhibitors | | | | |
| *MCL1* | | Amplification | | | | Tubulins,CDK4/6 inhibitor,sorafenib | | | | |
| *MDM2* | | Amplification | | | | Nutlin, MDM2 inhibitors | | | | |
| *MDM4* | | Amplification | | | | MDM4 inhibitors | | | | |
| *MITF* | | Mutation | | | | Vemurafenib, Dabrafenib, RAF inhibitors | | | | |
| *MLL* | | Rearrangement | | | | HDAC Inhibitors | | | | |
| *MPL* | | Mutation | | | | Ruxolitinib, JAK2 inhibitors | | | | |
| *MYD88* | | Mutation | | | | BTK inhibitors | | | | |
| *NF1* | | Inactivation | | | | PI3K/AKT/mTOR inhibitors,RAF inhibitors,MEK inhibitors | | | | |
| *NF2* | | Inactivation | | | | PI3K/AKT/mTOR inhibitors | | | | |
| *NOTCH1* | | Mutation; Rearrangement | | | | Notch Inhibitors | | | | |
| *NOTCH2* | | Mutation | | | | Notch Inhibitors | | | | |
| *NTRK3* | | Rearrangement | | | | PI3K/AKT/mTOR inhibitors,Dasatinib,src inhibitors,IGF1-R inhibitors | | | | |
| *PIK3R1* | | Mutations | | | | PI3K/AKT/mTOR inhibitors | | | | |
| *RAB35* | | Mutation | | | | WNT inhibitors | | | | |
| *RAF1* | | Mutation; Amplification; Rearrangement | | | | Sorafenib,RAF inhibitors | | | | |
| *RARA* | | Rearrangement; mutation | | | | ATRA, Arsenic | | | | |
| *RHEB* | | mutation | | | | mTOR inhibitors | | | | |
| *ROS1* | | Rearrangement | | | | Crizotinib | | | | |
| *SMARCA4* | | Inactivation | | | | HDAC inhibitor | | | | |
| *SMARCB1* | | Inactivation | | | | CDK inhibitors,Vismodegib, Hedgehog inhibitors,HDAC inhibitors | | | | |
| *SYK* | | Mutation | | | | SYK inhibitors | | | | |
| *TMPRSS2* | | Rearrangement | | | | PARP Inhibitors | | | | |
| *TP53* | | Inactivation | | | | Wee1 inhibitors,Chk1 inhibitors,kevetrin,APR-246,nutlins ,gene therapy | | | | |
| *EPHA5* | | Mutation; Rearrangement | | | | Dasatinib,Ephrin inhibitors | | | | |
| *FGFR4* | | Mutations; Rearrangement | | | | FGFR Inhibitors Ponatinib | | | | |
| *AXL* | | Mutation | | | | SGI-7079 BG8324 | | | | |
| *CDK13* | | Mutation | | | | Alvocidib | | | | |
| *JAK1* | | mutation | | | | ruxolitinib | | | | |
| *IL6ST* | | mutation | | | | tocilixumab | | | | |
| *ROCK1* | | mutation | | | | RKI-1447 | | | | |
| *SRC* | | mutation | | | | saracatinib,dasatinib,Bosutinib | | | | |
| *TGFBR1* | | mutation | | | | LY2157299 | | | | |
| *NOTCH3* | | mutation | | | | MK0752 | | | | |
| *NOTCH4* | | mutation | | | | MK0752 | | | | |
| *PRKAA1* | | mutation | | | | mTOR inhibitor | | | | |
| *FCGR2A* | | germline mutation | | | | Trastuzumab,Rituximab | | | | |
| *FCGR3A* | | germline mutation | | | | Trastuzumab,Rituximab | | | | |
| *PSMB1* | | mutation | | | | Bortezomib | | | | |
| *PSMB5* | | mutation | | | | Bortezomib,Carfilzomib | | | | |
| *DDR1* | | mutation | | | | Nilotinib | | | | |
| *PTCH2* | | Mutations | | | | Vismodegib, hedgehog inhibitors | | | | |
| *FCGR2B* | | germline mutation | | | | Rituximab | | | | |
| *C1QA* | | germline mutation | | | | Rituximab | | | | |
| *C1S* | | mutation | | | | Rituximab | | | | |
| *BCR* | | fusion | | | | imatinib,Bosutinib,Nilotinib, | | | | |
| *CSF1R* | | mutation | | | | Sunitinib,imatinib | | | | |
| *BCL2L11* | | germline | | | | Gefitinib | | | | |
| *EPHA2* | | mutation | | | | mTOR inhibitor | | | | |
| *HDAC1* | | mutation | | | | HDAC inhibitor | | | | |
| *HDAC4* | | mutation | | | | HDAC inhibitor | | | | |
| *RPS6KB1* | | expressing,mutation | | | | mTOR inhibitor | | | | |
| *FLT1* | | expressing | | | | VEGF/VEGFR inhibitor | | | | |
| *FLT4* | | expressing,snp | | | | VEGF/VEGFR inhibitor、sunitinib | | | | |
| *PIK3R2* | | Mutation | | | | PI3K inhibitors | | | | |
| *STAT1* | | Mutation or rearrangement | | | | JAK–STAT inhibitors | | | | |
| *STAT3* | | Mutation or rearrangement | | | | JAK–STAT inhibitors | | | | |
| *EPOR* | | Rearrangement | | | | JAK–STAT inhibitors | | | | |
| *IL7R* | | Mutation | | | | JAK–STAT inhibitors | | | | |
| *CDK8* | | Amplification, mutation, deletion or rearrangement | | | | CDK inhibitors | | | | |
| *HGF* | | mutation | | | | HGF inhibitor | | | | |
| *CHEK1* | | mutation ,overexpressing | | | | CHK inhibitor | | | | |
| *CHEK2* | | mutation ,overexpressing | | | | CHK inhibitor | | | | |
| *AURKB* | | Amplification,overexpressing | | | | AURKB inhibitor | | | | |
| *MS4A1* | | positive | | | | Rituximab,Ibritumomab,Tositumomab,Obinutuzumab | | | | |
| *ABL2* | | copy number | | | | Dasatinib | | | | |
| *MED12* | | Inactivation | | | | EGFR inhibitors | | | | |
| *VHL* | | Inactivation | | | | VEGF inhibitors | | | | |
| *XPO1* | | Mutation | | | | SINE agents | | | | |
| *TOP1* | | Mutation | | | | Camptothecin | | | | |
| *VEGFA* | | SNP | | | | sunitinib | | | | |
| *FAT1* | | Mutation | | | | Rigosertib | | | | |
| *ROBO3* | | Mutation | | | | Rigosertib | | | | |
| *ACIN1* | | Mutation | | | | Rigosertib | | | | |
| *ABCC11* | | Mutation | | | | Rigosertib | | | | |
| *CDH23* | | Mutation | | | | Rigosertib | | | | |
| *HCLS1* | | Mutation | | | | Rigosertib | | | | |
| *ERCC1* | | Mutation | | | | Satraplatin | | | | |
| *XRCC1* | | Mutation | | | | Satraplatin | | | | |
| *BTK* | | Mutation | | | | ibrutinib | | | | |
| **3. genes implicated in major cancer-related signaling pathways** | | | | | | | | | |  |
| **Gene Symbol** | | | | **Core pathway** | | | | | |  |
| *AKT1* | | | | PI3K signaling | | | | | |  |
| *AKT3* | | | | PI3K signaling | | | | | |  |
| *ALK* | | | | RTK signaling | | | | | |  |
| *APC* | | | | Wnt/b-catenin signaling | | | | | |  |
| *ARID1A* | | | | Chromatin SWI/SNF complex | | | | | |  |
| *ARID1B* | | | | Chromatin SWI/SNF complex | | | | | |  |
| *ARID2* | | | | Chromatin SWI/SNF complex | | | | | |  |
| *ATM* | | | | Genome integrity | | | | | |  |
| *BCL2L1* | | | | Apoptosis regulation | | | | | |  |
| *BCL2L2* | | | | Cell cycle | | | | | |  |
| *BCL6* | | | | Translation regulation | | | | | |  |
| *BCORL1* | | | | Translation regulation | | | | | |  |
| *BLM* | | | | Genome integrity | | | | | |  |
| *BRAF* | | | | MAPK signaling | | | | | |  |
| *CCND1* | | | | Cell cycle | | | | | |  |
| *CCNE1* | | | | Cell cycle | | | | | |  |
| *CDKN1B* | | | | Cell cycle | | | | | |  |
| *CDKN2A* | | | | Cell cycle | | | | | |  |
| *CDKN2B* | | | | Cell cycle | | | | | |  |
| *CHD2* | | | | Chromatin SWI/SNF complex | | | | | |  |
| *CHD4* | | | | Chromatin SWI/SNF complex | | | | | |  |
| *CRKL* | | | | RTK signaling | | | | | |  |
| *CTNNA1* | | | | APC Signaling | | | | | |  |
| *CTNNB1* | | | | Wnt/b-catenin signaling | | | | | |  |
| *DNMT3A* | | | | Epigenetics DNA methylation | | | | | |  |
| *DOT1L* | | | | Chromatin regulation | | | | | |  |
| *EGFR* | | | | RTK signaling | | | | | |  |
| *EP300* | | | | Chromatin histone acetyltransferases | | | | | |  |
| *EPHB1* | | | | RTK signaling | | | | | |  |
| *EPHB2* | | | | RTK signaling | | | | | |  |
| *ERBB2* | | | | RTK signaling | | | | | |  |
| *ERG* | | | | Transcription factor lineage dependency or oncogene | | | | | |  |
| *EZH2* | | | | Chromatin histone methyltransferases | | | | | |  |
| *FAM46C* | | | | Translation/protein homeostasis/ubiquitination | | | | | |  |
| *FANCA* | | | | Genome integrity | | | | | |  |
| *FANCD2* | | | | Genome integrity | | | | | |  |
| *FBXW7* | | | | Translation/protein homeostasis/ubiquitination | | | | | |  |
| *FGFR1* | | | | RTK signaling | | | | | |  |
| *FGFR2* | | | | RTK signaling | | | | | |  |
| *GATA3* | | | | Transcription factor | | | | | |  |
| *GSK3B* | | | | APC Signaling | | | | | |  |
| *HIF1A* | | | | Translation regulation | | | | | |  |
| *IDH1* | | | | Metabolism | | | | | |  |
| *IDH2* | | | | Metabolism | | | | | |  |
| *IGF2* | | | | MAPK signaling | | | | | |  |
| *IKZF1* | | | | Translation regulation | | | | | |  |
| *INHBA* | | | | TGF-b signaling | | | | | |  |
| *IRF4* | | | | Translation regulation | | | | | |  |
| *IRS2* | | | | RTK signaling | | | | | |  |
| *JAK2* | | | | RTK signaling | | | | | |  |
| *JUN* | | | | MAPK signaling | | | | | |  |
| *KAT6A* | | | | Chromatin regulation | | | | | |  |
| *KDM5A* | | | | Chromatin histone demethylases | | | | | |  |
| *KDM5C* | | | | Chromatin histone demethylases | | | | | |  |
| *KEAP1* | | | | Other signaling | | | | | |  |
| *KRAS* | | | | MAPK signaling | | | | | |  |
| *LYN* | | | | RTK signaling | | | | | |  |
| *MAP2K1* | | | | MAPK signaling | | | | | |  |
| *MAP2K4* | | | | Other signaling | | | | | |  |
| *MAP3K1* | | | | Other signaling | | | | | |  |
| *MCL1* | | | | Apoptosis regulation | | | | | |  |
| *MDM2* | | | | Genome integrity | | | | | |  |
| *MED12* | | | | Transcription factor | | | | | |  |
| *MEF2B* | | | | Translation regulation | | | | | |  |
| *MET* | | | | RTK signaling | | | | | |  |
| *MITF* | | | | Transcription factor lineage dependency or oncogene | | | | | |  |
| *MLL* | | | | Chromatin histone methyltransferases | | | | | |  |
| *MLL2* | | | | Chromatin histone methyltransferases | | | | | |  |
| *MLL3* | | | | Chromatin histone methyltransferases | | | | | |  |
| *MYC* | | | | Transcription factor | | | | | |  |
| *MYD88* | | | | NF-kB signaling | | | | | |  |
| *NF1* | | | | MAPK signaling | | | | | |  |
| *NFE2L2* | | | | Transcription factor | | | | | |  |
| *NKX2-1* | | | | Transcription factor lineage dependency or oncogene | | | | | |  |
| *NOTCH1* | | | | Notch signaling | | | | | |  |
| *NOTCH2* | | | | Notch signaling | | | | | |  |
| *NOTCH3* | | | | Notch signaling | | | | | |  |
| *NRAS* | | | | MAPK signaling | | | | | |  |
| *NSD1* | | | | Chromatin histone methyltransferases | | | | | |  |
| *NTRK2* | | | | MAPK signaling | | | | | |  |
| *PAK3* | | | | MAPK signaling | | | | | |  |
| *PBRM1* | | | | Chromatin SWI/SNF complex | | | | | |  |
| *PDGFRA* | | | | RTK signaling | | | | | |  |
| *PDK1* | | | | PI3K signaling | | | | | |  |
| *PIK3C2B* | | | | PI3K signaling | | | | | |  |
| *PIK3CA* | | | | PI3K signaling | | | | | |  |
| *PIK3CB* | | | | PI3K signaling | | | | | |  |
| *PIK3R1* | | | | PI3K signaling | | | | | |  |
| *PML* | | | | Cell cycle | | | | | |  |
| *POLE* | | | | Genome integrity | | | | | |  |
| *PRKDC* | | | | PI3K signaling | | | | | |  |
| *PRPF40B* | | | | Splicing | | | | | |  |
| *PTEN* | | | | PI3K signaling | | | | | |  |
| *RAC1* | | | | Other signaling | | | | | |  |
| *RAD51* | | | | Cell cycle | | | | | |  |
| *RET* | | | | RTK signaling | | | | | |  |
| *ROS1* | | | | RTK signaling | | | | | |  |
| *RPTOR* | | | | mTOR signaling | | | | | |  |
| *RUNX1* | | | | Transcription factor | | | | | |  |
| *RUNX1T1* | | | | Translation regulation | | | | | |  |
| *SF3B1* | | | | Transcription factor | | | | | |  |
| *SLIT2* | | | | Other signaling | | | | | |  |
| *SMAD2* | | | | TGF-b signaling | | | | | |  |
| *SMAD4* | | | | TGF-b signaling | | | | | |  |
| *SMARCA4* | | | | Chromatin SWI/SNF complex | | | | | |  |
| *SOX10* | | | | APC Signaling | | | | | |  |
| *SOX9* | | | | Transcription factor | | | | | |  |
| *SPOP* | | | | Translation/protein homeostasis/ubiquitination | | | | | |  |
| *SRSF2* | | | | Splicing | | | | | |  |
| *STAT4* | | | | STAT Signaling | | | | | |  |
| *STK11* | | | | mTOR signaling | | | | | |  |
| *SUFU* | | | | Hh Signaling | | | | | |  |
| *TET2* | | | | Epigenetics DNA hydroxymethylation | | | | | |  |
| *TGFBR2* | | | | TGF-b signaling | | | | | |  |
| *TOP2A* | | | | Translation regulation | | | | | |  |
| *TP53* | | | | Genome integrity | | | | | |  |
| *TSC1* | | | | mTOR signaling | | | | | |  |
| *TSC2* | | | | mTOR signaling | | | | | |  |
| *TYR* | | | | RTK signaling | | | | | |  |
| *U2AF1* | | | | Splicing | | | | | |  |
| *U2AF2* | | | | Splicing | | | | | |  |
| *WISP3* | | | | Wnt/b-catenin signaling | | | | | |  |
| *ZBTB2* | | | | Genome integrity | | | | | |  |
| *ZRSR2* | | | | Splicing | | | | | |  |
| *RPS14* | | | | Genome integrity | | | | | |  |
| **4. genes identified in the findings of the TCGA network covering 12 cancer types** | | | | | | | | |  | |
| **Gene Symbol** | **Name** | | | | **Tumor Types** | | **Cancer Syndrome** | |  | |
| *ACVR1B* | activin A receptor, type IB | | | | colorectal | |  | |  | |
| *AKT1* | v-akt murine thymoma viral oncogene homolog 1 | | | | breast, colorectal, ovarian, NSCLC | |  | |  | |
| *APC* | adenomatous polyposis of the colon gene | | | | colorectal, pancreatic, desmoid, hepatoblastoma, glioma, other CNS | | adenomatous polyposis coli; Turcot syndrome | |  | |
| *AR* | androgen receptor | | | | colorectal,Stomach | |  | |  | |
| *ARFRP1* | ADP-ribosylation factor related protein 1 | | | | colorectal | |  | |  | |
| *ARID1A* | AT rich interactive domain 1A (SWI-like) | | | | clear cell ovarian carcinoma, RCC, breast | |  | |  | |
| *ASXL1* | additional sex combs like 1 | | | | MDS, CMML | |  | |  | |
| *ATM* | ataxia telangiectasia mutated | | | | T-PLL | | ataxia-telangiectasia | |  | |
| *ATR* | ATR serine/threonine kinase | | | | endometrial, gastric | | familial cutaneous telangiectasia and cancer sydrome | |  | |
| *ATRX* | alpha thalassemia/mental retardation syndrome X-linked | | | | pancreatic neuroendocrine tumours, paediatric GBM | |  | |  | |
| *AXIN2* | axin 2 | | | | colorectal | | oligodontia-colorectal cancer syndrome | |  | |
| *BAK1* | BCL2-antagonist/killer 1 | | | | Endometrium | |  | |  | |
| *BAP1* | BRCA1 associated protein-1 (ubiquitin carboxy-terminal hydrolase) | | | | uveal melanoma, breast, NSCLC, RCC | |  | |  | |
| *BRAF* | v-raf murine sarcoma viral oncogene homolog B1 | | | | melanoma, colorectal, papillary thyroid, borderline ovarian, NSCLC, cholangiocarcinoma, pilocytic astrocytoma, Spitzoid tumour, pancreas acinar carcinoma, melanocytic nevus, prostate, gastric | |  | |  | |
| *BRCA1* | familial breast/ovarian cancer gene 1 | | | | ovarian | | hereditary breast/ovarian cancer | |  | |
| *BRCA2* | familial breast/ovarian cancer gene 2 | | | | breast, ovarian, pancreatic | | hereditary breast/ovarian cancer | |  | |
| *C11orf30* |  | | | | ovarian,breast | |  | |  | |
| *CBFB* | core-binding factor, beta subunit | | | | AML | |  | |  | |
| *CBLB* | Cas-Br-M (murine) ecotropic retroviral transforming sequence b | | | | AML | |  | |  | |
| *CCND1* | cyclin D1 | | | | CLL, B-ALL, breast | |  | |  | |
| *CD274* | CD274 molecule | | | | tumor | |  | |  | |
| *CD79B* | CD79b molecule, immunoglobulin-associated beta | | | | DLBCL, WM | |  | |  | |
| *CDH1* | cadherin 1, type 1, E-cadherin (epithelial) (ECAD) | | | | lobular breast, gastric | | familial gastric carcinoma | |  | |
| *CDK12* | cyclin-dependent kinase 12 | | | | serous ovarian | |  | |  | |
| *CDKN1A* | cyclin-dependent kinase inhibitor 1A | | | | Urinary tract,Skin | |  | |  | |
| *CDKN1B* | cyclin-dependent kinase inhibitor 1B (p27, Kip1) | | | | breast, small intestine neuroendocrine tumours | | multiple endocrine neoplasia type IV | |  | |
| *CDKN2A* | cyclin-dependent kinase inhibitor 2A (p16(INK4a)) gene | | | | melanoma, multiple other tumour types | | familial malignant melanoma | |  | |
| *CDKN2C* | cyclin-dependent kinase inhibitor 2C (p18, inhibits CDK4) | | | | glioma, MM | |  | |  | |
| *CEBPA* | CCAAT/enhancer binding protein (C/EBP), alpha | | | | AML, MDS | |  | |  | |
| *CHEK2* | CHK2 checkpoint homolog (S. pombe) | | | | breast | | familial breast cancer | |  | |
| *CTCF* | CCCTC-binding factor (zinc finger protein) | | | | Endometrium,colorectal | |  | |  | |
| *CTNNB1* | catenin (cadherin-associated protein), beta 1 | | | | colorectal, ovarian, hepatoblastoma, pleomorphic salivary gland adenoma, other tumour types | |  | |  | |
| *CUL3* | cullin 3 | | | | Endometrium | |  | |  | |
| *DICER1* | dicer 1, ribonuclease type III | | | | sex cord-stromal tumour, TGCT, embryonal rhabdomyosarcoma, pleuropulmonary blastoma, pituitary blastoma, Wilms tumour, thyroid cancer, other tumour types | | familial pleuropulmonary blastoma or DICER1 syndrome | |  | |
| *DNMT3A* | DNA (cytosine-5-)-methyltransferase 3 alpha | | | | AML | |  | |  | |
| *EGFR* | epidermal growth factor receptor (erythroblastic leukemia viral (v-erb-b) oncogene homolog, avian) | | | | glioma, NSCLC | | familial lung cancer | |  | |
| *EP300* | 300 kd E1A-Binding protein gene | | | | colorectal, breast, pancreatic, AML, ALL, DLBCL | |  | |  | |
| *EPHA3* | EPH receptor A3 | | | | Skin,colorectal,Stomach,Lung | |  | |  | |
| *EPHA7* | EPH receptor A7 | | | | Skin,colorectal,Stomach,Lung | |  | |  | |
| *EPHB6* | EPH receptor B6 | | | | colorectal,lung,breast | |  | |  | |
| *ERBB4* | erb-b2 receptor tyrosine kinase 4 | | | | colorectal,lung | |  | |  | |
| *ETV6* | ets variant gene 6 (TEL oncogene) | | | | congenital fibrosarcoma, multiple different leukaemia and lymphoma tumour types including ALL, secretory breast, MDS | |  | |  | |
| *EZH2* | enhancer of zeste homolog 2 | | | | DLBCL | |  | |  | |
| *FBXW7* | F-box and WD-40 domain protein 7 (archipelago homolog, Drosophila) | | | | colorectal, endometrial, T-ALL | |  | |  | |
| *FGFR2* | fibroblast growth factor receptor 2 | | | | gastric, NSCLC, endometrial | |  | |  | |
| *FGFR3* | fibroblast growth factor receptor 3 | | | | bladder, MM, T-cell lymphoma | |  | |  | |
| *FLT3* | fms-related tyrosine kinase 3 | | | | AML, ALL | |  | |  | |
| *FOXP1* | forkhead box P1 | | | | ALL | |  | |  | |
| *GAB2* | GRB2-associated binding protein 2 | | | | Endometrium,colorectal | |  | |  | |
| *GABRA6* | gamma-aminobutyric acid (GABA) A receptor, alpha 6 | | | | skin,Stomach | |  | |  | |
| *GATA3* | GATA binding protein 3 | | | | breast | |  | |  | |
| *GATA4* | GATA binding protein 4 | | | | colorectal,Stomach | |  | |  | |
| *GATA6* | GATA binding protein 4 | | | | Peritoneum | |  | |  | |
| *GLI1* | GLI family zinc finger 1 | | | | breast | |  | |  | |
| *GNA13* | guanine nucleotide binding protein (G protein), alpha 13 | | | | breast | |  | |  | |
| *GPR124* | G protein-coupled receptor 124 | | | | breast | |  | |  | |
| *GRIN2A* | glutamate receptor, ionotropic, N-methyl D-aspartate 2A | | | | melanoma | |  | |  | |
| *GRM3* | glutamate receptor, metabotropic 3 | | | | skin | |  | |  | |
| *HGF* | hepatocyte growth factor | | | | Peritoneum,Skin | |  | |  | |
| *HSD3B1* | hydroxy-delta-5-steroid dehydrogenase, 3 beta- and steroid delta-isomerase 1 | | | | prostate cancer | |  | |  | |
| *HSP90AA1* | heat shock protein 90kDa alpha (cytosolic), class A member 1 | | | | NHL | |  | |  | |
| *IDH1* | isocitrate dehydrogenase 1 (NADP+), soluble | | | | glioblastoma | |  | |  | |
| *IDH2* | isocitrate dehydrogenase 2 (NADP+), mitochondrial | | | | glioblastoma | |  | |  | |
| *IKBKE* | inhibitor of kappa light polypeptide gene enhancer in B-cells, kinase epsilon | | | | breast | |  | |  | |
| *INPP4B* | inositol polyphosphate-4-phosphatase, type II | | | | Endometrium | |  | |  | |
| *IRF2* | interferon regulatory factor 2 | | | | stomach | |  | |  | |
| *KDM5C* | lysine (K)-specific demethylase 5C (JARID1C) | | | | clear cell renal carcinoma | |  | |  | |
| *KDM6A* | lysine (K)-specific demethylase 6A, UTX | | | | renal, oesophageal SCC, MM | |  | |  | |
| *KEAP1* | kelch-like ECH-associated protein 1 | | | | lung | |  | |  | |
| *KEL* | Kell blood group, metallo-endopeptidase | | | | Skin,Testis | |  | |  | |
| *KIF5B* | kinesin family member 5B | | | | NSCLC, Spitzoid tumour | |  | |  | |
| *KIT* | v-kit Hardy-Zuckerman 4 feline sarcoma viral oncogene homolog | | | | GIST, AML, TGCT, mastocytosis, mucosal melanoma | | familial gastrointestinal stromal tumour | |  | |
| *KLHL6* | kelch-like family member 6 | | | | Cervix,lung | |  | |  | |
| *KRAS* | v-Ki-ras2 Kirsten rat sarcoma 2 viral oncogene homolog | | | | pancreatic, colorectal, lung, thyroid, AML, other tumour types | |  | |  | |
| *LMO1* | LIM domain only 1 (rhombotin 1) (RBTN1) | | | | T-ALL, neuroblastoma | |  | |  | |
| *LRP1B* | low density lipoprotein receptor-related protein 1B | | | | colorectal, liver,lung,skin,stomach | |  | |  | |
| *LZTR1* | leucine-zipper-like transcription regulator 1 | | | | Endometrium | |  | |  | |
| *MAGI2* | membrane associated guanylate kinase, WW and PDZ domain containing 2 | | | | colorectal, liver,lung,skin,stomach | |  | |  | |
| *MAP2K4* | mitogen-activated protein kinase kinase 4 | | | | pancreatic, breast, colorectal | |  | |  | |
| *MAP3K1* | mitogen-activated protein kinase kinase kinase 1, E3 ubiquitin protein ligase | | | | luminal A breast | |  | |  | |
| *MC1R* | melanocortin 1 receptor (alpha melanocyte stimulating hormone receptor) | | | | melanoma | |  | |  | |
| *MLL2* | lysine (K)-specific methyltransferase 2B | | | | breast,colorectal | |  | |  | |
| *MLL3* | lysine (K)-specific methyltransferase 2C | | | | colorectal | |  | |  | |
| *MTOR* | mechanistic target of rapamycin (serine/threonine kinase) | | | | breast | |  | |  | |
| *MYCL1* | v-myc avian myelocytomatosis viral oncogene lung carcinoma derived homolog | | | | Urinary tract,lung | |  | |  | |
| *MYCN* | v-myc myelocytomatosis viral related oncogene, neuroblastoma derived (avian) | | | | neuroblastoma | |  | |  | |
| *NCOR1* | nuclear receptor corepressor 1 | | | | breast | |  | |  | |
| *NF1* | neurofibromatosis type 1 gene | | | | neurofibroma, glioma | | neurofibromatosis type 1 | |  | |
| *NFE2L2* | nuclear factor (erythroid-derived 2)-like 2 (NRF2) | | | | NSCLC, HNSCC | |  | |  | |
| *NFKBIA* | nuclear factor of kappa light polypeptide gene enhancer in B-cells inhibitor, alpha | | | | lung | |  | |  | |
| *NOTCH1* | Notch homolog 1, translocation-associated (Drosophila) (TAN1) | | | | T-ALL, breast, bladder, skin SCC, lung SCC, head and neck SCC | |  | |  | |
| *NPM1* | nucleophosmin (nucleolar phosphoprotein B23, numatrin) | | | | NHL, APL, AML | |  | |  | |
| *NRAS* | neuroblastoma RAS viral (v-ras) oncogene homolog | | | | melanoma, MM, AML, thyroid | |  | |  | |
| *NSD1* | nuclear receptor binding SET domain protein 1 | | | | AML | |  | |  | |
| *NUP93* | nucleoporin | | | | colorectal | |  | |  | |
| *PARK2* | parkin RBR E3 ubiquitin protein ligase | | | | colorectal,Stomach | |  | |  | |
| *PBRM1* | polybromo 1 | | | | clear cell renal carcinoma, breast | |  | |  | |
| *PDGFRA* | platelet-derived growth factor, alpha-receptor | | | | GIST, idiopathic hypereosinophilic syndrome, paediatric glioblastoma | |  | |  | |
| *PHF6* | PHD finger protein 6 | | | | ETP ALL | |  | |  | |
| *PIK3CA* | phosphoinositide-3-kinase, catalytic, alpha polypeptide | | | | colorectal, gastric, glioblastoma, breast | |  | |  | |
| *PIK3CG* | phosphatidylinositol-4,5-bisphosphate 3-kinase, catalytic subunit gamma | | | | colorectal, lung,skin | |  | |  | |
| *PIK3R1* | phosphoinositide-3-kinase, regulatory subunit 1 (alpha) | | | | glioblastoma, ovarian, colorectal | |  | |  | |
| *PLCG2* | phospholipase C, gamma 2 (phosphatidylinositol-specific) | | | | colorectal, lung,skin | |  | |  | |
| *POLD1* | polymerase (DNA directed), delta 1, catalytic subunit | | | | colorectal,stomach | |  | |  | |
| *PPP2R1A* | protein phosphatase 2, regulatory subunit A, alpha | | | | clear cell ovarian carcinoma | |  | |  | |
| *PREX2* | phosphatidylinositol-3,4,5-trisphosphate-dependent Rac exchange factor 2 | | | | colorectal,breast | |  | |  | |
| *PRKAR1A* | protein kinase, cAMP-dependent, regulatory, type I, alpha (tissue specific extinguisher 1) | | | | papillary thyroid | | Carney complex | |  | |
| *PRKCI* | protein kinase C, iota | | | | Cervix,lung | |  | |  | |
| *PRSS8* | protease, serine, 8 | | | | ovarian | |  | |  | |
| *PTEN* | phosphatase and tensin homolog gene | | | | glioma, prostate, endometrial | | Cowden syndrome, Bannayan-Riley-Ruvalcaba syndrome | |  | |
| *PTPN11* | protein tyrosine phosphatase, non-receptor type 11 | | | | JMML, AML, MDS | |  | |  | |
| *PTPRD* | protein tyrosine phosphatase, receptor type, D | | | | colorectal, lung,skin | |  | |  | |
| *QKI* | KH domain containing, RNA binding | | | | lung | |  | |  | |
| *RANBP2* | RAN binding protein 2 | | | | inflammatory myofibroblastic tumour | |  | |  | |
| *RB1* | retinoblastoma gene | | | | retinoblastoma, sarcoma, breast, small cell lung carcinoma | | familial retinoblastoma | |  | |
| *RBM10* | RNA binding motif protein 10 | | | | Peritoneum | |  | |  | |
| *RICTOR* | RPTOR independent companion of MTOR, complex 2 | | | | Cervix,lung | |  | |  | |
| *RUNX1* | runt-related transcription factor 1 (AML1) | | | | AML, preB- ALL, T-ALL | |  | |  | |
| *SDHA* | succinate dehydrogenase complex, subunit A, flavoprotein (Fp) | | | | lung,Cervix | |  | |  | |
| *SETBP1* | SET binding protein 1 | | | | aCML, sAML, MDS/MPN-U, CMML, JMML | | Schinzel-Giedion syndrome | |  | |
| *SETD2* | SET domain containing 2 | | | | clear cell renal carcinoma | |  | |  | |
| *SF1* | splicing factor 1 | | | | colorectal | |  | |  | |
| *SF3B1* | splicing factor 3b, subunit 1, 155kDa | | | | myelodysplastic syndrome | |  | |  | |
| *SMAD2* | SMAD family member 2 | | | | colorectal | |  | |  | |
| *SMAD3* | SMAD family member 3 | | | | colorectal | |  | |  | |
| *SMAD4* | SMAD family member 4 | | | | colorectal, pancreatic, small intestine | | juvenile polyposis | |  | |
| *SNCAIP* | synuclein, alpha interacting protein | | | | skin | |  | |  | |
| *SOX2* | SRY (sex determining region Y)-box 2 | | | | NSCLC, oesophageal squamous carcinoma | |  | |  | |
| *SOX9* | SRY (sex determining region Y)-box 9 | | | | colorectal | |  | |  | |
| *SPEN* | spen family transcriptional repressor | | | | SMZL, adenoid cystic carcinoma, DLBCL | |  | |  | |
| *SPOP* | speckle-type POZ protein | | | | prostate, serous endometrial | |  | |  | |
| *SPRY4* | sprouty RTK signaling antagonist 4 | | | | Tumor of genital tract | |  | |  | |
| *SPTA1* | spectrin, alpha, erythrocytic 1 | | | | lung,colorectal,stomach,liver | |  | |  | |
| *STAG2* | stromal antigen 2 | | | | bladder carcinoma, glioblastoma, melanoma, Ewing's sarcoma, myeloid neoplasms | |  | |  | |
| *STK11* | serine/threonine kinase 11 gene (LKB1) | | | | NSCLC, pancreatic | | Peutz-Jeghers syndrome | |  | |
| *SUZ12* | suppressor of zeste 12 homolog (Drosophila) | | | | endometrial stromal tumour | |  | |  | |
| *TAF1* | TAF1 RNA polymerase II, TATA box binding protein (TBP)-associated factor | | | | endometrial | |  | |  | |
| *TBX3* | T-box 3 | | | | breast | |  | |  | |
| *TERC* | telomerase RNA component | | | | lung,cervical cancer | |  | |  | |
| *TET2* | tet oncogene family member 2 | | | | MDS | |  | |  | |
| *TFG* | TRK-fused gene | | | | papillary thyroid, ALCL, NSCLC, extraskeletal myxoid chondrosarcoma | |  | |  | |
| *TGFBR2* | transforming growth factor, beta receptor II | | | | colorectal,Stomach | |  | |  | |
| *TNFRSF14* | tumor necrosis factor receptor superfamily, member 14 (herpesvirus entry mediator) | | | | follicular lymphoma | |  | |  | |
| *TP53* | tumor protein p53 | | | | breast, colorectal, lung, sarcoma, adrenocortical, glioma, Spitzoid tumour, multiple other tumour types | | Li-Fraumeni syndrome | |  | |
| *TRRAP* | transformation/transcription domain-associated protein | | | | melanoma | |  | |  | |
| *U2AF1* | U2 small nuclear RNA auxiliary factor 1 | | | | CLL, MDS | |  | |  | |
| *VHL* | von Hippel-Lindau syndrome gene | | | | renal, haemangioma, pheochromocytoma | | Von Hippel-Lindau syndrome | |  | |
| *WT1* | Wilms tumour 1 gene | | | | Wilms tumour, desmoplastic small round cell tumour | | Denys-Drash syndrome, Frasier syndrome, familial Wilms tumour | |  | |
| *ZNF217* | zinc finger protein 217 | | | | breast,colorectal,Stomach | |  | |  | |
| *ZNF703* | zinc finger protein 703 | | | | breast | |  | |  | |

Supplementary Table 3. Detailed sequencing data for 18 patients

| **Sample ID** | **Type** | **Total effective reads (M)** | **Fraction of reads uniquely mapped to genome** | **Total uniquely mapped reads (M)** | **Fraction of uniquely mapped reads on target** | **Uniquely mapped reads on target (M)** | **Average sequencing depth on target** | **Coverage of target region** | **Fraction of target covered with at least 20x** | **Target region size (M)** |
| --- | --- | --- | --- | --- | --- | --- | --- | --- | --- | --- |
| 1 | peripheral blood cells | 36.58 | 95.75% | 19.82 | 54.46% | 10.8 | 525 | 99.65% | 98.87% | 1.61 |
| 1-baseline | plasma | 84.17 | 95.44% | 75.16 | 16.39% | 12.32 | 603 | 99.93% | 98.98% | 1.61 |
| 1-C2 | plasma | 38.85 | 98.76% | 37.65 | 67.18% | 25.29 | 1185 | 99.76% | 99.37% | 1.61 |
| 2 | peripheral blood cells | 12.13 | 98.41% | 11.73 | 70.03% | 8.22 | 353 | 99.76% | 99.06% | 1.76 |
| 2-baseline | plasma | 76.34 | 95.17% | 68.15 | 10.04% | 6.84 | 323 | 99.94% | 98.97% | 1.61 |
| 2-C2 | plasma | 62.2 | 97.71% | 59.53 | 21.72% | 12.93 | 595 | 99.89% | 99.33% | 1.61 |
| 2-C4 | plasma | 34.24 | 98.60% | 32.78 | 48.97% | 16.05 | 679 | 99.97% | 99.69% | 1.76 |
| 3 | peripheral blood cells | 16.36 | 96.97% | 15.23 | 56.03% | 8.53 | 419 | 99.57% | 94.69% | 1.61 |
| 3-baseline | plasma | 77.29 | 95.20% | 69.15 | 13.09% | 9.05 | 435 | 99.94% | 99.01% | 1.61 |
| 3-C2 | plasma | 39.26 | 98.55% | 37.54 | 45.99% | 17.27 | 725 | 99.95% | 99.72% | 1.76 |
| 3-C4 | plasma | 63.85 | 97.79% | 61.5 | 32.65% | 20.08 | 942 | 99.89% | 99.35% | 1.61 |
| 3-C6 | plasma | 79.64 | 97.37% | 74.91 | 31.07% | 23.27 | 989 | 99.97% | 99.67% | 1.76 |
| 4 | peripheral blood cells | 25.01 | 97.70% | 23.77 | 51.61% | 12.27 | 541 | 99.86% | 99.28% | 1.76 |
| 4-baseline | plasma | 67.84 | 95.44% | 60.91 | 17.29% | 10.53 | 516 | 99.84% | 98.91% | 1.61 |
| 4-C2 | plasma | 30.59 | 98.74% | 29.43 | 58.61% | 17.25 | 797 | 99.79% | 99.38% | 1.61 |
| 4-C6 | plasma | 69.6 | 97.68% | 66.38 | 28.61% | 18.99 | 808 | 99.97% | 99.67% | 1.76 |
| 4-C8 | plasma | 62.8 | 98.24% | 60.03 | 26.21% | 15.74 | 662 | 99.93% | 99.58% | 1.76 |
| 5-baseline | plasma | 88.38 | 97.66% | 84.61 | 35.75% | 30.25 | 1300 | 99.96% | 99.68% | 1.76 |
| 5-C2 | plasma | 55.5 | 98.16% | 52.78 | 15.35% | 8.1 | 336 | 99.94% | 99.60% | 1.76 |
| 6 | peripheral blood cells | 16.44 | 96.11% | 14.85 | 48.63% | 7.22 | 326 | 99.68% | 98.76% | 1.76 |
| 6-baseline | plasma | 66.92 | 97.50% | 63.65 | 18.44% | 11.74 | 489 | 99.98% | 99.60% | 1.76 |
| 6-C2 | plasma | 56.7 | 96.73% | 52.5 | 15.40% | 8.08 | 338 | 99.98% | 99.64% | 1.76 |
| 6-C6 | plasma | 46.91 | 98.02% | 44.61 | 19.37% | 8.64 | 359 | 99.96% | 99.56% | 1.76 |
| 6-C8 | plasma | 19.56 | 99.39% | 18.73 | 51.99% | 9.74 | 404 | 99.91% | 99.59% | 1.76 |
| 7 | peripheral blood cells | 15.19 | 96.39% | 13.74 | 50.39% | 6.92 | 312 | 99.72% | 98.92% | 1.76 |
| 7-baseline | plasma | 74.04 | 97.40% | 70.21 | 13.49% | 9.47 | 390 | 99.98% | 99.55% | 1.76 |
| 7-C2 | plasma | 55.51 | 97.79% | 52.03 | 26.77% | 13.93 | 581 | 99.96% | 99.55% | 1.76 |
| 7-C4 | plasma | 88.91 | 96.04% | 82.91 | 25.27% | 20.95 | 899 | 99.95% | 99.59% | 1.76 |
| 8 | peripheral blood cells | 16.27 | 98.25% | 15.36 | 59.35% | 9.12 | 404 | 99.79% | 99.17% | 1.76 |
| 8-baseline | plasma | 46.21 | 97.85% | 42.98 | 44.65% | 19.19 | 841 | 99.91% | 99.55% | 1.76 |
| 8-C2 | plasma | 70.45 | 98.07% | 66.04 | 47.48% | 31.35 | 1380 | 99.91% | 99.49% | 1.76 |
| 9-baseline | plasma | 70.7 | 97.79% | 67.34 | 21.02% | 14.15 | 592 | 99.97% | 99.65% | 1.76 |
| 9-C2 | plasma | 60.85 | 98.15% | 58.17 | 24.75% | 14.4 | 607 | 99.93% | 99.57% | 1.76 |
| 10 | peripheral blood cells | 13.43 | 96.90% | 12.2 | 64.70% | 7.89 | 356 | 99.79% | 99.06% | 1.76 |
| 10-baseline | plasma | 79.38 | 97.13% | 75.2 | 12.60% | 9.48 | 391 | 99.97% | 99.62% | 1.76 |
| 10-C2 | plasma | 59.95 | 96.77% | 55.49 | 18.37% | 10.2 | 426 | 99.98% | 99.76% | 1.76 |
| 11 | peripheral blood cells | 17.05 | 98.57% | 16.38 | 55.77% | 9.14 | 401 | 99.75% | 98.85% | 1.76 |
| 11-baseline | plasma | 61.16 | 98.08% | 58.34 | 34.27% | 19.99 | 867 | 99.96% | 99.24% | 1.76 |
| 11-C2 | plasma | 91.22 | 98.47% | 87.62 | 34.37% | 30.11 | 1294 | 99.94% | 99.62% | 1.76 |
| 12 | peripheral blood cells | 18.35 | 97.80% | 17.57 | 52.15% | 9.16 | 396 | 99.75% | 99.09% | 1.76 |
| 12-baseline | plasma | 87.22 | 97.73% | 83.33 | 23.70% | 19.75 | 833 | 99.94% | 99.76% | 1.76 |
| 12-C2 | plasma | 57.96 | 96.05% | 53.53 | 23.90% | 12.79 | 551 | 99.98% | 99.66% | 1.76 |
| 13 | peripheral blood cells | 16.97 | 98.37% | 16.33 | 64.47% | 10.53 | 455 | 99.74% | 99.15% | 1.76 |
| 13-baseline | plasma | 45.79 | 98.70% | 43.75 | 42.74% | 18.7 | 777 | 99.94% | 99.69% | 1.76 |
| 13-C2 | plasma | 47.89 | 97.40% | 44.71 | 21.93% | 9.8 | 407 | 99.96% | 99.52% | 1.76 |
| 14 | peripheral blood cells | 19.26 | 96.21% | 17.83 | 42.82% | 7.64 | 376 | 99.56% | 98.63% | 1.61 |
| 14-baseline | plasma | 93.28 | 97.59% | 89.94 | 40.24% | 36.19 | 1723 | 99.88% | 99.19% | 1.61 |
| 14-C2 | plasma | 84.71 | 94.66% | 75.93 | 19.97% | 15.16 | 750 | 99.84% | 98.61% | 1.61 |
| 15 | peripheral blood cells | 17.36 | 97.92% | 16.63 | 52.78% | 8.78 | 380 | 99.73% | 99.06% | 1.76 |
| 15-baseline | plasma | 30.89 | 98.69% | 29.43 | 41.67% | 12.26 | 508 | 99.92% | 99.65% | 1.76 |
| 15-C2 | plasma | 40.05 | 96.80% | 37.25 | 27.53% | 10.26 | 432 | 99.95% | 99.62% | 1.76 |
| 15-C4 | plasma | 71.77 | 96.62% | 66.97 | 17.30% | 11.59 | 483 | 99.95% | 99.60% | 1.76 |
| 15-C8 | plasma | 56.65 | 96.77% | 52.77 | 14.52% | 7.66 | 317 | 99.95% | 99.58% | 1.76 |
| 16 | peripheral blood cells | 15.05 | 99.36% | 14.3 | 72.92% | 10.43 | 463 | 99.85% | 99.26% | 1.76 |
| 16-baseline | plasma | 49.61 | 98.25% | 45.82 | 16.92% | 7.75 | 318 | 99.94% | 99.55% | 1.76 |
| 16-C2 | plasma | 6.62 | 99.02% | 6.25 | 59.87% | 3.74 | 158 | 99.86% | 98.89% | 1.76 |
| 17 | peripheral blood cells | 13.51 | 98.16% | 12.94 | 53.74% | 6.95 | 300 | 99.73% | 99.03% | 1.76 |
| 17-baseline | plasma | 84.19 | 97.81% | 80.46 | 22.62% | 18.2 | 760 | 99.94% | 99.70% | 1.76 |
| 17-C2 | plasma | 21.26 | 98.18% | 20 | 59.31% | 11.86 | 504 | 99.93% | 99.46% | 1.76 |
| 17-C4 | plasma | 74.93 | 96.52% | 70.09 | 26.40% | 18.51 | 789 | 99.93% | 99.52% | 1.76 |
| 17-C8 | plasma | 55.21 | 97.60% | 52.4 | 44.72% | 23.43 | 990 | 99.98% | 99.67% | 1.76 |
| 17-C10 | plasma | 80.16 | 97.13% | 76.34 | 26.66% | 20.35 | 867 | 99.98% | 99.52% | 1.76 |
| 18 | peripheral blood cells | 14.66 | 98.79% | 14.16 | 70.48% | 9.98 | 429 | 99.78% | 99.20% | 1.76 |
| 18-baseline | plasma | 212.03 | 95.96% | 190.95 | 14.06% | 26.84 | 1181 | 100.00% | 99.81% | 1.76 |
| 18-C2 | plasma | 77.12 | 97.69% | 74.03 | 25.35% | 18.76 | 871 | 99.92% | 99.33% | 1.61 |
| 18-C6 | plasma | 90.76 | 97.43% | 86.5 | 13.75% | 11.9 | 489 | 99.96% | 99.73% | 1.76 |
| 18-C8 | plasma | 44.06 | 96.92% | 40.97 | 34.02% | 13.94 | 600 | 99.97% | 99.68% | 1.76 |
| 18-C10 | plasma | 64.62 | 96.25% | 60.41 | 24.19% | 14.61 | 622 | 99.93% | 99.54% | 1.76 |

Supplementary Table 4. Somatic copy number variants (CNVs) identified in 52 plasma samples

| **Patient ID** | **NO. of CNVs** | **Gene** | **Copy Number** | | | | | |
| --- | --- | --- | --- | --- | --- | --- | --- | --- |
|  |  |  | **Baseline** | **C2** | **C4** | **C6** | **C8** | **C10** |
| 1 | 1 | *DNMT1* | 0.6 | 0.6 | ND | ND | ND | ND |
| 2 | 3 | *ERBB2* | 2 | 3.4 | 3.4 |  |  |  |
|  |  | *CDK12* | 2 | 2 | 3.2 |  |  |  |
|  |  | *NFKBIA* | 2 | 2 | 1.2 |  |  |  |
| 3 | 6 | *ERBB2* | 2 | 2 | 4.4 | 5.6 |  |  |
|  |  | *CDK12* | 2 | 2 | 2 | 4.2 |  |  |
|  |  | *CDK4* | 2 | 2 | 2 | 4.8 |  |  |
|  |  | *RARA* | 2 | 2 | 2 | 4.2 |  |  |
|  |  | *EGFR* | 2 | 2 | 2 | 3.8 |  |  |
|  |  | *TOP2A* | 2 | 2 | 2 | 3.8 |  |  |
| 4 | 1 | *NFKBIA* | 2 | 0.8 | ND | 2 | 2 |  |
| 5 | 5 | *ERBB2* | 2 | 6.8 |  |  |  |  |
|  |  | *NFKBIA* | 1 | 2 |  |  |  |  |
|  |  | *CDK12* | 2 | 4.8 |  |  |  |  |
|  |  | *RPTOP* | 2 | 5 |  |  |  |  |
|  |  | *SPOP* | 2 | 5.2 |  |  |  |  |
| 6 | 2 | *ERBB2* | 2.8 | 2 | ND | 2 | 2 |  |
|  |  | *HLA-A* | 2 | 2 |  | 1.2 | 1 |  |
| 7 | 4 | *ERBB2* | 3.2 | 2 | 2.8 |  |  |  |
|  |  | *FGF3* | 9.8 | 2 | 2 |  |  |  |
|  |  | *FGF4* | 7.2 | 2 | 2 |  |  |  |
|  |  | *GAB2* | 4.6 | 2 | 2 |  |  |  |
| 8 | 1 | *ERBB2* | 11 | 19 |  |  |  |  |
| 9 |  | *MLL* | 0.6 | 0.6 |  |  |  |  |
| 10* | 0 | NA | NA | NA | ND | ND | ND | ND |
| 11 | 3 | *ERBB2* | 13 | 11.6 |  |  |  |  |
|  |  | *CDK12* | 2 | 9 |  |  |  |  |
|  |  | *FGFR1* | 6.2 | 6.4 |  |  |  |  |
| 12 | 1 | *ERBB2* | 3 | 3.2 |  |  |  |  |
| 13 | 1 | *ERBB2* | 2.6 | 2 |  |  |  |  |
| 14 | 5 | *CCND1* | 12 | 12 |  |  |  |  |
|  |  | *FGF19* | 17.4 | 15.6 |  |  |  |  |
|  |  | *FGF3* | 25.4 | 22.8 |  |  |  |  |
|  |  | *FGF4* | 21.8 | 19.8 |  |  |  |  |
|  |  | *GPR124* | 16 | 13.4 |  |  |  |  |
| 15 | 4 | *ERBB2* | 7.4 | 2 | 2 | ND | 2 |  |
|  |  | *CD79B* | 9.8 | 2 | 2 |  | 2 |  |
|  |  | *CDK12* | 7.4 | 2 | 2 |  | 2 |  |
|  |  | *SRSF2* | 2 | 2 | 1 |  | 2 |  |
| 16 | 3 | *ERBB2* | 4.2 | 2 | ND | ND | ND | ND |
|  |  | *CDK12* | 4.2 | 2 |  |  |  |  |
|  |  | *RPS6KB1* | 4 | 2 |  |  |  |  |
| 17 | 3 | *ERBB2* | 2 | 2 | 2 | ND | 2 | 3 |
|  |  | *NFKBIA* | 1 | 2 | 2 |  | 1 | 1 |
|  |  | *HLA-A* | 1 | 2 | 2 |  | 1 | 1 |
| 18 | 1 | *ERBB2* | 2.4 | 1.3 | ND | 1 | 2.3 | 1.8 |

Copy number ≥2.6 (CONTRA log-ratio ≥ 1.3) and ≤1 (CONTRA log-ratio ≤ 0.5) were determined as gain and loss, respectively.

NA-- Not Applicable; ND (Not Done) represents that samples collected at the corresponding time points were not subjected to sequencing.

* We failed to detect CNVs in the baseline and C2 plasma samples of Patient No. 10.

Supplementary Table 5. Non-silent SNV and small indels identified in 52 plasma samples

| **Patient** | **NO. of Mutations** | **Mutation** | **Mutation AF (%)** | | | | | |
| --- | --- | --- | --- | --- | --- | --- | --- | --- |
|  |  |  | **Baseline** | **C2** | **C4** | **C6** | **C8** | **C10** |
| 1 | 4 | BCR c.[559G>T] | 2.48 | 0.00 | ND | ND | ND | ND |
|  |  | KMT2D c.[58C>A] | 1.54 | 0.00 |  |  |  |  |
|  |  | PTPRD c.[1976C>A] | 1.06 | 0.00 |  |  |  |  |
|  |  | SF1 c.[1276C>T] | 0.00 | 1.54 |  |  |  |  |
| 2 | 5 | CEBPA c.[589_590insACCCGC] | 33.33 | 26.84 | 28.76 |  |  |  |
|  |  | KLHL6 c.[643G>A] | 0.00 | 1.00 | 0.00 |  |  |  |
|  |  | MSH6 c.[529C>T] | 0.00 | 1.10 | 0.00 |  |  |  |
|  |  | CROT c.[1152A>C] | 0.00 | 0.00 | 1.36 |  |  |  |
|  |  | DOT1L c.[967G>A] | 0.00 | 0.00 | 1.40 |  |  |  |
| 3 | 4 | PIK3CA c.[3140A>G] | 1.59 | 0.00 | 5.44 | 16.36 |  |  |
|  |  | SMARCB1 c.[1142C>T] | 0.00 | 1.14 | 0.00 | 0.00 |  |  |
|  |  | TP53 c.[375+2T>G] | 0.00 | 1.39 | 4.88 | 13.36 |  |  |
|  |  | CEBPA c.[862C>T] | 0.00 | 0.00 | 1.12 | 0.00 |  |  |
| 4 | 8 | AR c.[1369_1371delGGC] | 21.71 | 22.83 | ND | 15.90 | 26.92 |  |
|  |  | CEBPA c.[589_590insACCCGC] | 21.62 | 31.79 |  | 31.17 | 29.49 |  |
|  |  | PIK3CA c.[3140A>G] | 1.52 | 1.32 |  | 6.93 | 4.89 |  |
|  |  | KLF4 c.[497G>A] | 0.00 | 0.00 |  | 1.29 | 0.00 |  |
|  |  | TP53 c.[318C>G] | 0.00 | 0.00 |  | 2.53 | 2.33 |  |
|  |  | ATRX c.[2435C>T] | 0.00 | 0.00 |  | 0.00 | 1.21 |  |
|  |  | DOT1L c.[3803G>A] | 0.00 | 0.00 |  | 0.00 | 1.10 |  |
|  |  | FAT3 c.[8161A>T] | 0.00 | 0.00 |  | 0.00 | 0.80 |  |
| 5 | 25 | BRCA2 c.[5024G>A] | 47.37 | 46.29 |  |  |  |  |
|  |  | EXT2 c.[890G>A] | 48.21 | 45.96 |  |  |  |  |
|  |  | FLT3 c.[657delA] | 69.41 | 0.00 |  |  |  |  |
|  |  | JAK2 c.[2982delT] | 20.76 | 0.00 |  |  |  |  |
|  |  | MSH5 c.[2249C>T] | 48.56 | 46.18 |  |  |  |  |
|  |  | MUTYH c.[1471delA] | 9.43 | 0.00 |  |  |  |  |
|  |  | PARP1 c.[1910delA] | 27.29 | 0.00 |  |  |  |  |
|  |  | PIK3CA c.[3140A>G] | 1.63 | 15.86 |  |  |  |  |
|  |  | PIK3R1 c.[238delA] | 50.24 | 0.00 |  |  |  |  |
|  |  | TSC2 c.[1873T>C] | 49.20 | 42.52 |  |  |  |  |
|  |  | TSHR c.[2168delA] | 41.00 | 0.00 |  |  |  |  |
|  |  | ARAF c.[217G>A] | 0.00 | 1.80 |  |  |  |  |
|  |  | ATR c.[4217C>T] | 0.00 | 2.87 |  |  |  |  |
|  |  | BCL6 c.[1739G>A] | 0.00 | 4.32 |  |  |  |  |
|  |  | CDK12 c.[3271C>G] | 0.00 | 4.48 |  |  |  |  |
|  |  | CHD2 c.[2652C>G] | 0.00 | 2.62 |  |  |  |  |
|  |  | CYLD c.[2401G>A] | 0.00 | 6.63 |  |  |  |  |
|  |  | ERBB2 c.[3235G>A] | 0.00 | 1.53 |  |  |  |  |
|  |  | FLT1 c.[2082G>C] | 0.00 | 9.77 |  |  |  |  |
|  |  | KMT2C c.[4873G>C] | 0.00 | 1.82 |  |  |  |  |
|  |  | MTOR c.[6286G>C] | 0.00 | 1.80 |  |  |  |  |
|  |  | PDGFRB c.[2877G>C] | 0.00 | 2.47 |  |  |  |  |
|  |  | SMARCA1 c.[766C>T] | 0.00 | 2.16 |  |  |  |  |
|  |  | SOX9 c.[1251G>C] | 0.00 | 9.80 |  |  |  |  |
|  |  | TP53 c.[811G>T] | 0.00 | 9.09 |  |  |  |  |
| 6 | 6 | ROBO2 c.[1457A>G] | 1.96 | 1.86 | ND | 1.83 | 1.60 |  |
|  |  | FAT3 c.[7393T>A] | 0.00 | 0.93 |  | 0.00 | 0.00 |  |
|  |  | KDM6A c.[3883T>A] | 0.00 | 1.11 |  | 0.00 | 0.00 |  |
|  |  | SETD2 c.[7572T>A] | 0.00 | 1.05 |  | 0.00 | 0.00 |  |
|  |  | STAG2 c.[1419A>T] | 0.00 | 1.15 |  | 0.00 | 0.00 |  |
|  |  | RPS14 c.[218C>A] | 0.00 | 0.00 |  | 0.00 | 2.27 |  |
| 7 | 3 | RPL22 c.[37delA] | 3.93 | 0.00 | 0.00 |  |  |  |
|  |  | TP53 c.[497C>G] | 3.06 | 0.00 | 1.53 |  |  |  |
|  |  | RAD51C c.[5G>T] | 0.00 | 0.00 | 1.46 |  |  |  |
| 8 | 4 | ARID1B c.[1379_1381delCGG] | 5.83 | 0.00 |  |  |  |  |
|  |  | IRF4 c.[31G>T] | 9.18 | 16.45 |  |  |  |  |
|  |  | PML c.[851G>C] | 10.34 | 20.36 |  |  |  |  |
|  |  | ROS1 c.[6316G>A] | 9.61 | 15.85 |  |  |  |  |
| 9 | 20 | ATM c.[5788G>C] | 5.56 | 7.09 |  |  |  |  |
|  |  | ATM c.[8246A>T] | 49.63 | 61.67 |  |  |  |  |
|  |  | BRCA2 c.[7135G>A] | 47.53 | 43.37 |  |  |  |  |
|  |  | DOCK2 c.[4046G>T] | 4.79 | 9.14 |  |  |  |  |
|  |  | ERCC3 c.[974G>A] | 47.12 | 44.92 |  |  |  |  |
|  |  | ESR1 c.[1610A>C] | 4.36 | 7.93 |  |  |  |  |
|  |  | MET c.[890A>G] | 3.48 | 6.56 |  |  |  |  |
|  |  | NFE2L3 c.[1244C>G] | 2.52 | 4.62 |  |  |  |  |
|  |  | NOTCH4 c.[227delA] | 37.68 | 0.00 |  |  |  |  |
|  |  | PALB2 c.[3256C>T] | 60.51 | 64.27 |  |  |  |  |
|  |  | PRX c.[3107C>T] | 4.62 | 6.00 |  |  |  |  |
|  |  | SMAD3 c.[261C>G] | 4.13 | 5.90 |  |  |  |  |
|  |  | SRSF1 c.[401G>A] | 3.71 | 3.25 |  |  |  |  |
|  |  | TP53 c.[392A>G] | 8.12 | 10.83 |  |  |  |  |
|  |  | TYR c.[113C>T] | 56.08 | 59.82 |  |  |  |  |
|  |  | USP9X c.[4507G>C] | 2.84 | 5.70 |  |  |  |  |
|  |  | APCDD1 c.[609C>A] | 0.00 | 2.14 |  |  |  |  |
|  |  | GRM3 c.[1868G>A] | 0.00 | 1.22 |  |  |  |  |
|  |  | IKBKB c.[1689-1G>C] | 0.00 | 1.03 |  |  |  |  |
|  |  | NOTCH1 c.[4319_4320insC] | 0.00 | 15.99 |  |  |  |  |
| 10 | 4 | ASXL1 c.[1927delG] | 3.44 | 0.00 | ND | ND | ND | ND |
|  |  | FAT3 c.[5792delT] | 0.00 | 0.47 |  |  |  |  |
|  |  | KDM5A c.[1491-1delG] | 0.00 | 0.31 |  |  |  |  |
|  |  | MDM4 c.[608delT] | 0.00 | 0.59 |  |  |  |  |
| 11 | 20 | AMER1 c.[16G>T] | 1.00 | 4.01 |  |  |  |  |
|  |  | CDK12 c.[848C>T] | 0.36 | 0.00 |  |  |  |  |
|  |  | FAT3 c.[6787G>A] | 0.91 | 1.48 |  |  |  |  |
|  |  | GNAS c.[543G>C] | 36.20 | 35.84 |  |  |  |  |
|  |  | KMT2C c.[1013-2A>G] | 1.31 | 0.00 |  |  |  |  |
|  |  | NAV3 c.[4271A>G] | 1.01 | 0.00 |  |  |  |  |
|  |  | NFE2L2 c.[125G>C] | 1.01 | 0.00 |  |  |  |  |
|  |  | RNASEL c.[505G>T] | 2.10 | 2.14 |  |  |  |  |
|  |  | TFG c.[530T>A] | 19.58 | 17.25 |  |  |  |  |
|  |  | ATRX c.[3100C>T] | 0.00 | 3.02 |  |  |  |  |
|  |  | AXIN1 c.[1613A>C] | 0.00 | 2.60 |  |  |  |  |
|  |  | CYP17A1 c.[655C>T] | 0.00 | 0.67 |  |  |  |  |
|  |  | EPHA5 c.[2962G>T] | 0.00 | 0.65 |  |  |  |  |
|  |  | HNF1A c.[649G>T] | 0.00 | 0.58 |  |  |  |  |
|  |  | KDM6A c.[3821C>T] | 0.00 | 2.60 |  |  |  |  |
|  |  | MED12 c.[3745C>A] | 0.00 | 5.61 |  |  |  |  |
|  |  | MSH2 c.[1742T>G] | 0.00 | 8.26 |  |  |  |  |
|  |  | SF1 c.[482G>A] | 0.00 | 1.45 |  |  |  |  |
|  |  | SYK c.[1091A>G] | 0.00 | 0.64 |  |  |  |  |
|  |  | VHL c.[430G>A] | 0.00 | 1.18 |  |  |  |  |
| 12 | 12 | ASPSCR1 c.[791C>T] | 2.42 | 2.51 |  |  |  |  |
|  |  | CDK12 c.[3724C>T] | 1.78 | 1.30 |  |  |  |  |
|  |  | FAT3 c.[5792delT] | 0.45 | 0.00 |  |  |  |  |
|  |  | FLI1 c.[1019G>A] | 3.21 | 0.00 |  |  |  |  |
|  |  | KAT6A c.[3390A>C] | 1.72 | 1.26 |  |  |  |  |
|  |  | KDM5A c.[1491-1delG] | 0.67 | 0.00 |  |  |  |  |
|  |  | NOTCH4 c.[2288A>T] | 2.04 | 0.00 |  |  |  |  |
|  |  | PRKCA c.[877G>A] | 1.99 | 1.85 |  |  |  |  |
|  |  | ROBO1 c.[4178C>A] | 2.96 | 1.58 |  |  |  |  |
|  |  | SPEN c.[1595A>G] | 1.24 | 0.00 |  |  |  |  |
|  |  | TP53 c.[706T>A] | 4.35 | 1.46 |  |  |  |  |
|  |  | KAT6A c.[5470A>T] | 0.00 | 0.88 |  |  |  |  |
| 13 | 10 | BRIP1 c.[587A>G] | 1.56 | 0.00 |  |  |  |  |
|  |  | NCOA1 c.[709G>A] | 7.88 | 1.97 |  |  |  |  |
|  |  | NCOA2 c.[3868C>T] | 1.34 | 1.31 |  |  |  |  |
|  |  | NUP93 c.[700T>G] | 1.07 | 0.00 |  |  |  |  |
|  |  | PIK3CA c.[3140A>G] | 39.04 | 12.78 |  |  |  |  |
|  |  | PIK3R1 c.[889G>A] | 7.88 | 0.00 |  |  |  |  |
|  |  | TOP2A c.[1627-1G>C] | 32.10 | 12.60 |  |  |  |  |
|  |  | TP53 c.[659A>G] | 20.26 | 7.81 |  |  |  |  |
|  |  | DOT1L c.[1854G>T] | 0.00 | 1.09 |  |  |  |  |
|  |  | PIK3CA c.[296delT] | 0.00 | 0.24 |  |  |  |  |
| 14 | 9 | CTNNB1 c.[788delT] | 0.16 | 0.00 |  |  |  |  |
|  |  | ERBB3 c.[3143delT] | 0.25 | 0.00 |  |  |  |  |
|  |  | ERCC3 c.[777A>T] | 0.46 | 0.00 |  |  |  |  |
|  |  | KAT6A c.[4561G>A] | 0.39 | 0.00 |  |  |  |  |
|  |  | KDM5A c.[1010G>C] | 2.33 | 2.15 |  |  |  |  |
|  |  | NSD1 c.[7643delT] | 0.32 | 0.00 |  |  |  |  |
|  |  | PIK3CA c.[1035T>A] | 36.02 | 51.05 |  |  |  |  |
|  |  | SETBP1 c.[2591C>T] | 0.61 | 0.00 |  |  |  |  |
|  |  | BCL6 c.[1427C>A] | 0.00 | 1.64 |  |  |  |  |
| 15 | 16 | FANCA c.[3418A>T] | 1.28 | 0.00 | 0.00 | ND | 0.00 |  |
|  |  | FGFR3 c.[1138G>A] | 1.21 | 0.00 | 0.00 |  | 0.00 |  |
|  |  | GRIN2A c.[1310G>A] | 1.80 | 0.00 | 0.00 |  | 0.00 |  |
|  |  | NAV3 c.[5696C>G] | 2.51 | 0.00 | 0.00 |  | 0.00 |  |
|  |  | NCOA2 c.[1516C>T] | 10.42 | 0.00 | 0.00 |  | 0.00 |  |
|  |  | PDK1 c.[1264G>A] | 1.26 | 0.00 | 0.00 |  | 0.00 |  |
|  |  | RARB c.[1078C>T] | 9.52 | 0.00 | 0.00 |  | 0.00 |  |
|  |  | TSC2 c.[3968C>T] | 1.12 | 0.00 | 0.00 |  | 0.00 |  |
|  |  | CYLD c.[2612A>T] | 0.00 | 1.29 | 0.00 |  | 0.00 |  |
|  |  | ERBB3 c.[1187A>T] | 0.00 | 1.30 | 0.00 |  | 0.00 |  |
|  |  | GSK3B c.[608+2T>A] | 0.00 | 1.42 | 0.00 |  | 0.00 |  |
|  |  | IL2RG c.[272A>T] | 0.00 | 1.21 | 0.00 |  | 0.00 |  |
|  |  | JAK1 c.[3298A>T] | 0.00 | 1.21 | 0.00 |  | 0.00 |  |
|  |  | SOX2 c.[676T>A] | 0.00 | 1.02 | 0.00 |  | 0.00 |  |
|  |  | MLH3 c.[3149T>A] | 0.00 | 0.00 | 1.02 |  | 0.00 |  |
|  |  | MTOR c.[1077C>A] | 0.00 | 0.00 | 0.00 |  | 2.31 |  |
| 16 | 1 | DDR1 c.[1994C>T] | 0.00 | 3.11 | ND | ND | ND | ND |
| 17 | 11 | BCL6 c.[682C>T] | 1.03 | 0.00 | 0.00 | ND | 0.00 | 0.00 |
|  |  | BRCA2 c.[7403T>A] | 0.00 | 0.84 | 0.00 |  | 0.00 | 0.00 |
|  |  | FLT1 c.[1574C>A] | 0.00 | 0.92 | 0.00 |  | 0.00 | 0.00 |
|  |  | TP53 c.[833C>G] | 0.00 | 0.56 | 0.00 |  | 0.62 | 0.00 |
|  |  | TP53 c.[672+1G>T] | 0.00 | 0.00 | 0.00 |  | 0.00 | 1.59 |
|  |  | FAT3 c.[8746G>A] | 0.00 | 0.00 | 0.00 |  | 0.71 | 1.00 |
|  |  | ATM c.[2764G>C] | 0.00 | 0.00 | 0.00 |  | 0.00 | 0.64 |
|  |  | FGFR2 c.[2425C>A] | 0.00 | 0.00 | 0.00 |  | 0.00 | 1.20 |
|  |  | RAD50 c.[3085G>T] | 0.00 | 0.00 | 0.00 |  | 0.00 | 1.19 |
|  |  | RAD50 c.[568G>A] | 0.00 | 0.00 | 0.00 |  | 0.00 | 2.04 |
|  |  | PIK3CA c.[3140A>G] | 0.00 | 0.00 | 0.00 |  | 1.41 | 0.00 |
|  |  | PIK3CA c.[1637A>G] | 0.00 | 0.00 | 0.00 |  | 3.01 | 9.79 |
| 18 | 22 | ARID1A c.[6709G>A] | 3.70 | 0.00 | ND | 0.00 | 8.83 | 4.06 |
|  |  | CDH1 c.[1387G>C] | 1.72 | 0.00 |  | 0.00 | 0.00 | 0.00 |
|  |  | ELF3 c.[281G>A] | 5.51 | 0.00 |  | 1.06 | 7.05 | 1.81 |
|  |  | FGFR3 c.[1138G>A] | 1.71 | 0.00 |  | 0.00 | 0.00 | 0.00 |
|  |  | PDGFRA c.[430G>T] | 2.97 | 0.00 |  | 0.00 | 0.00 | 0.00 |
|  |  | RAF1 c.[863C>A] | 1.56 | 0.00 |  | 0.00 | 0.00 | 0.00 |
|  |  | SF3A1 c.[284G>A] | 1.57 | 0.00 |  | 0.00 | 0.00 | 0.00 |
|  |  | NOTCH1 c.[4067G>A] | 0.00 | 1.06 |  | 0.00 | 0.00 | 0.00 |
|  |  | CHD2 c.[209C>T] | 0.00 | 0.00 |  | 1.37 | 0.00 | 1.06 |
|  |  | LYN c.[1229C>T] | 0.00 | 0.00 |  | 2.21 | 0.00 | 0.00 |
|  |  | APC c.[4111A>C] | 0.00 | 0.00 |  | 0.00 | 2.96 | 1.69 |
|  |  | ATR c.[1095A>T] | 0.00 | 0.00 |  | 0.00 | 1.25 | 0.00 |
|  |  | BARD1 c.[1969C>G] | 0.00 | 0.00 |  | 0.00 | 4.70 | 0.00 |
|  |  | BARD1 c.[400A>T] | 0.00 | 0.00 |  | 0.00 | 1.04 | 0.00 |
|  |  | CTNNA1 c.[1828G>T] | 0.00 | 0.00 |  | 0.00 | 1.56 | 0.00 |
|  |  | ERBB2 c.[2264T>C] | 0.00 | 0.00 |  | 0.00 | 1.10 | 1.34 |
|  |  | FCGR2A c.[94G>T] | 0.00 | 0.00 |  | 0.00 | 1.33 | 0.00 |
|  |  | SIN3A c.[388C>T] | 0.00 | 0.00 |  | 0.00 | 2.15 | 0.00 |
|  |  | XPO1 c.[1711G>A] | 0.00 | 0.00 |  | 0.00 | 5.91 | 0.00 |
|  |  | FGF14 c.[608C>A] | 0.00 | 0.00 |  | 0.00 | 0.00 | 1.40 |
|  |  | KMT2A c.[433-2A>C] | 0.00 | 0.00 |  | 0.00 | 0.00 | 1.33 |
|  |  | PIK3CA c.[1624G>A] | 4.60 | 0.00 |  | 1.35 | 12.24 | 4.92 |
|  |  | PTEN c.[511C>T] | 0.00 | 0.00 |  | 0.00 | 0.00 | 1.46 |

ND (Not Done) represents that samples collected at the corresponding time points were not subjected to sequencing.
